# Metabolomic profiling of *Bacillus velezensis* B13 and unveiling its antagonistic potential for the sustainable management of rice sheath blight

**DOI:** 10.3389/fpls.2025.1554867

**Published:** 2025-07-25

**Authors:** Sirivella Naveena, Chellappan Gopalakrishnan, Rajendran Logeshwari, Muthurajan Raveendran, Ramamoorthy Pushpam, Paranthaman Lakshmidevi

**Affiliations:** ^1^ Department of Plant Pathology, Tamil Nadu Agricultural University, Coimbatore, Tamil Nadu, India; ^2^ Senior Research Coordinator, IFFCO- Nanoventions Private Limited, Coimbatore, Tamil Nadu, India; ^3^ Directorate of Research, Tamil Nadu Agricultural University, Coimbatore, Tamil Nadu, India; ^4^ Department of Rice, Tamil Nadu Agricultural University, Coimbatore, Tamil Nadu, India; ^5^ Institute of Agriculture, Tamil Nadu Agricultural University, Kumulur, Trichy, Tamil Nadu, India

**Keywords:** *Bacillus velezensis*, *Rhizoctonia solani*, secondary metabolites, molecular docking, defense genes expression

## Abstract

Sheath blight disease is accountable for substantial loss in rice production worldwide. Endophytic bacteria are exploited as biocontrol agents due to their effectiveness in antagonizing a wide range of phytopathogens through a multifaceted approach. In the present study, the potentiality of deploying endophytic bacteria for the sustainable management of rice sheath blight was investigated. Over 40 bacterial endophytes were obtained and screened for their antagonistic activity against *Rhizoctonia solani* by a dual-culture assay. Among them, *B. velezensis* B13 exhibited higher mycelial inhibition (77.33%) against *R. solani*. A scanning electron microscopic study of the interaction of *R. solani* with B13 revealed distorted and deformed mycelia of *R. solani*. An analysis of secondary metabolites produced by *B. velezensis* B13 at their zone of interaction with *R. solani* confirmed the presence of various bioactive compounds of an antifungal and antimicrobial nature. A molecular docking study revealed that the compound 3′,8,8′-Trimethoxy-3-piperidyl-2,2′-binaphthalene-1,1′,4,4′-tetrone exhibited the highest binding affinity for Actin like protein (−7.6 kcal/mol), β-1,3 glucan synthase (−7.7 kcal/mol), Pectinesterase (−4.2 kcal/mol) and Polygalacturonase (−6.5 kcal/mol) protein targets of *R. solani* compared to the commercial fungicide carbendazim. *In vivo* experiments also proved the efficacy of *B. velezensis* B13 in suppressing rice sheath blight disease reduction upto 16.8± 0.2 besides enhancing the growth of the plant. Furthermore, *B. velezensis* B13 upregulated the expression of rice transcription factors and defense genes, *viz*., WRKY, PR1, PAL, LOX, FLS2 and CERK1, by several folds related to the inoculated and healthy control, leading to the suppression of *R. solani*. Our results suggest that *B. velezensis* (B13) could be a potential candidate for developing a bioconsortia for the sustainable management of rice sheath blight.

## Introduction

Sheath blight of rice, caused by *Rhizoctonia solani*, is a destructive disease hindering rice production. Anastomosis group AG1-IA of *R. solani* is the dominant group infecting rice and is considered the primary causal agent of sheath blight, a serious disease impacting rice yield globally (Chen et al., 2023). AG1-IA exhibits high strain variation, including differences in virulence, genetic diversity and host adaptation. Multiple pathotypes and haplotypes have been identified within AG1-IA populations, reflecting its substantial genetic variability and adaptability, which pose challenge for breeding resistant rice cultivars (Zheng et al., 2013). The disease causes significant annual yield loss, ranging from 10% to 30%, and it is estimated to increase up to 50% in the coming years (Jamali et al., 2020). To date, only a few rice varieties resistant to sheath blight have been identified. Although several cultivars have exhibited varying levels of resistance, no variety has displayed complete immunity to the disease. The screening of resistant varieties against rice sheath blight is still in progress (Abbas et al., 2023). Commercial fungicides have been exploited for controlling the disease, but they are expensive and leave chemical residues in food materials, leading to health and environmental problems. An eco-friendly method for controlling the plant disease is the use of biocontrol agents, which is a sustainable agricultural practice (Kumar et al., 2020). Endophytic microorganisms are now widely used as biocontrol agents in agriculture due to their innate ability to colonize and reside within plant tissues, boosting their physiological and developmental processes. They also help overcome biotic and abiotic stresses encountered by the host plant (Wani et al., 2015).

Identifying potential endophytes for promoting plant growth and controlling diseases requires exploration of a range of bacterial endophytes found in landraces and rice varieties. Several research works have documented the isolation of bacterial endophytes of rice from traditional varieties of rice and wild accessions (Elbeltagy et al., 2000). Various approaches, including culture-based and culture-independent methods, have been employed to isolate endophytes from plant tissues, ovule and seed endosphere to determine their plant growth-promoting activities (Hardoim et al., 2012; Walitang et al., 2017).

Several endophytic bacterial genera, including *Bacillus*, *Strenotrophomonas*, *Pseudomonas*, *Burkholderia*, *Enterobacter*, *Micrococcus* and *Serratia*, are well known for producing lytic enzymes, such as chitinase, protease and β-glucanase, which are accountable for the degradation of the fungal cell wall during antagonistic interactions (Khare et al., 2018). Endophytic bacteria play a crucial role in promoting plant growth and minimizing pathogen infection through direct and indirect mechanisms. Their direct impact is primarily due to their antagonistic behavior against the pathogen by competing with and parasitizing them, exhibiting antibiosis and producing extracellular digestive enzymes. In contrast, the indirect impact is through the activation of plant defense mechanisms in response to various diseases (Soliman et al., 2022).

Endophytic *Bacillus* species have been investigated to combat sheath blight disease as they possess beneficial properties, such as stimulating plant growth and eliciting an immune response (Nagendran et al., 2014; Islam et al., 2020; Zheng et al., 2021). These bacteria can produce various antimicrobial compounds, *viz*., siderophores (bacillibactin), polyketides (bacillaene) and lipopeptides (fengycin, surfactin, bacillomycin D and iturin) (Nicholson, 2002). It is imperative to identify these secondary metabolites and their related antimicrobial nature to ascertain the biological control activity of *Bacillus* species. *Bacillus* spp. acts as plant safeguards against phytopathogens through the induction of systemic resistance and subsequent upregulation of the plant defense-linked genes (Seo et al., 2012; Rais et al., 2017). In the current investigation, the endophytic bacteria *B. velezensis* B13 was evaluated for its antagonistic potential, secondary metabolite production, determining the potential of the metabolites used in silico docking studies, and its efficacy for inducing the defense system against rice sheath blight disease.

## Materials and methods

### Collection and isolation of *Rhizocontia solani*

The plants showing typical sheath blight symptoms were collected during 2021–2022 from various rice growing regions of Tamil Nadu. The samples were brought to the laboratory and washed under running water to eliminate dirt particles and blot dried. A lesion of 2–3 mm portion of the advancing region was carefully excised; the surface was sterilized with 1% sodium hypochlorite (NaOCl) for 1 min and then rinsed thrice in sterile distilled water. The sterilized leaf tissues were dried with sterile filter paper and then transferred to a Petri plate containing potato dextrose agar (PDA) supplemented with streptomycin sulphate (PCT1120 Himedia, Mumbai, Maharashtra, India), followed by incubation at 28 ± 2°C for 5–7 days. The pure culture of isolates was transferred to the potato dextrose agar (PDA) slants by the single hyphal tip method and maintained at 25 ± 2°C for further studies. A glycerol stock of all the isolates was also preserved at −80°C for long-term storage (Sandoval and Cumagun, 2019).

### Isolation of endophytic bacteria

For endophytic bacteria isolation, healthy rice plants and wild rice species grown in Paddy Breeding Station, Tamil Nadu Agricultural University, Coimbatore, were used (**Table 1**). Plant tissues, such as leaves, stems and roots, were collected in a polythene bag and were brought to the laboratory. One gram of tissue samples was surface sterilized using a 0.5% sodium hypochlorite solution. The samples were then washed three times with sterile distilled water and dried on sterile filter paper to ensure sterility. The samples were homogenized using a pestle and mortar with 1 mL of 0.9% peptone saline buffer. A total of 1 ml of tissue suspension was subjected to serial dilution, and 10^−4^–10^−5^ dilutions were inoculated on Luria Bertani agar (M1151, HIMEDIA) plates and incubated at 28 ± 2°C for bacterial growth. The morphologically different bacterial colonies were re-streaked on LB agar plates. The purified bacteria were maintained in an LB broth of 20% sterile glycerol and preserved at −80 °C for further studies (Kumar et al., 2020).

**Table 1:** List of endophytic bacterial isolates obtained from commercial rice varieties and wild rice species.

S. No.	Rice Varieties	Location	Source of Isolation	Name of Bacterial Endophytes
1	TN1	Paddy Breeding Station, TNAU, Coimbatore	Root	B1
			Stem	B2
			Stem	B3
			Root	B4
			Root	B5
2	*Oryza officinalis*	Paddy Breeding Station, TNAU, Coimbatore	Leaf	B6
			Leaf	B7
			Leaf	B8
3	*Oryza eichengari*	Paddy Breeding Station, TNAU, Coimbatore	Leaf	B9
			Leaf	B10
			Leaf	B11
4	*Oryza nivara*	Paddy Breeding Station, TNAU, Coimbatore	Leaf	B12
			Leaf	B13
			Leaf	B14
5	*Oryza australiensis*	Paddy Breeding Station, TNAU, Coimbatore	Leaf	B15
			Leaf	B16
6	CO 39	Paddy Breeding Station, TNAU, Coimbatore	Root	B17
			Leaf	B18
			Stem	B19
			Stem	B20
			Root	B21
			Leaf	B22
7	ADT 37	Wetland, TNAU, Coimbatore	Root	B23
			Leaf	B24
			Stem	B25
			Root	B26
			Leaf	B27
			Leaf	B28
			Leaf	B29
8	*Oryza rhizomatis*	Paddy Breeding Station, TNAU, Coimbatore	Leaf	B30
			Leaf	B31
			Leaf	B32
9	*Oryza alta*	Paddy Breeding Station, TNAU, Coimbatore	Leaf	B33
			Leaf	B34
			Leaf	B35
			Leaf	B36
10	*Oryza latifolia*	Paddy Breeding Station, TNAU, Coimbatore	Leaf	B37
			Leaf	B38
12	*Oryza minuta*	Paddy Breeding Station, TNAU, Coimbatore	Leaf	B39
			Leaf	B40

### *In vitro* screening of endophytic bacteria against *R. solani*

The antagonistic activity of forty endophytic bacterial isolates was tested *in vitro* against a virulent isolate of *R. solani* AG1-1A which was isolated from var. ADT 43 (NCBI accession No. OQ940459.1). A freshly grown mycelial disc (9 mm) of a five-day old culture of *R. solani* was placed on one side, 1 cm away from the edge of the sterilized Petri plate. The bacterial endophytes were streaked perpendicular to the mycelial disc on the other side of the Petri plate (Jamali et al., 2020). The experiment was performed with three replications for each bacterial isolate, and a control plate was maintained by inoculating the pathogen alone at the end of the Petri dish containing the PDA medium. The plates were then incubated for seven days at room temperature (28 ± 2°C). The efficacy of the endophytic bacteria against the pathogen was determined based on the size of their inhibition zone. The radial growth of the pathogen and percent inhibition relative to the control were calculated using the following formula (Shakeel et al., 2015):


$$\text{Percent of inhibition over control }\%\left(\text{I}\right)=\frac{\left(\text{C}-\text{T}\right)}{\text{C}}\times 100$$

where C is the mycelial growth of the pathogen in the control and T is the mycelial growth of the pathogen in the dual-culture treatment. On the basis of the higher percentage inhibition of *R. solani*, *Bacillus* strain B13 was selected for further studies.

### Molecular characterization of effective endophytic bacteria

For the molecular identification of strain B13, the 1.5 kb full-length 16S rRNA gene was amplified by polymerase chain reaction (PCR) with a universal forward and reverse primer: 27F (5′-GAGTTTGATCCTGGCTCAG-3′) and 1492R (5′- GGTTACCTTGTTACGACTT-3′). Polymerase chain reaction (PCR) was performed in a 25 µL reaction mixture that included 10 µL of master mix (RR310 EmeraldAmp), 1 µL of bacterial genomic DNA at a concentration of 20 ng, 1 µL of each primer at a concentration of 10 µM and 12 µL of sterilized deionized water. The PCR amplification conditions of the thermocycler (Nexus Gradient, Eppendorf, Hamburg, Germany) were as follows: an initial denaturation at 94°C for 5 min, followed by 35 cycles of denaturation at 94°C for 1 min; primer annealing at 55°C for 1 min; extension at 72°C for 40 s and final extension at 72°C for 10 min. The amplified 16S rRNA gene product was visualized on 1% agarose gel with a UV transilluminator, photographed using the gel documentation system and sequenced at Biokart India Pvt. Ltd., Bangalore, India. The sequence similarities were determined using Basic Local Alignment Search Tool (BLAST) analysis (https://www.ncbi.nlm.nih.gov). The sequences with the greatest homology and highest similarity were obtained from the NCBI GenBank database, and multiple sequence alignment was conducted using the ClustalW algorithm. A neighbor joining (NJ) phylogenetic tree was constructed with bootstrap test (1000 replicates) using MEGA 11 software (Kumar et al., 2018).

### Scanning electron microscopy

SEM analysis was performed to further validate the effect of the extracellular secondary metabolites of *Bacillus* strain B13 on *R. solani* (Zhu et al., 2023). A small piece of freshly grown mycelia of *R. solani* regarded as the control and the mycelia and interaction region with B13 in the dual-culture plate were cut using a sterile scalpel and placed in perforated capsules. The samples were then fixed in 1.5% glutaraldehyde in phosphate buffer for 4 h. After fixation, the mycelia were rinsed with 0.2 M sodium cacodylate buffer (pH 6.2) and dehydrated in a series of ethanol concentrations (30%, 50%, 70%, 80%, 90% and 100%) for 15 min each. The dehydrated mycelia were then mounted onto aluminum stubs using conductive double-sided carbon tape, followed by subsequent sputter coating with gold in a rotary vacuum pump for 40 s for complete and uniform coating over the sample surface. Finally, the morphological changes in the mycelium of the pathogen were observed under a scanning electron microscope.

### GC/MS analysis of secondary metabolites extracted from the zone of inhibition of *R. solani* and bacterial strain B13

The secondary metabolites produced by the highly efficient bacterial endophyte (B13) and their di-trophic interaction with *R. solani* were characterized through gas chromatography–mass spectrophometry (GC–MS). The bioactive compounds produced by strain B13 during their di-trophic interaction with *R. solani* in PDA from the zone of inhibition were extracted by excising the agar using a sterile scalpel. The excised agar was blended with HPLC-grade acetonitrile in a 1:4 ratio (5 g agar in 20 mL of HPLC grade acetonitrile). For homogenization, the mixture was sonicated twice for 30 s at 30% of the power of the sonicator. After homogenization, the samples were centrifuged and filtered to eliminate the solid particles. A vacuum flash evaporator (Roteva Equitron Make, Mumbai, India) was used for drying the samples. The final product was dissolved in 1 mL of HPLC-grade methanol following the removal of the eluent (Kumar et al., 2018). The variation in the secondary metabolite profile generated during the interaction of B13 with *R. solani* was analyzed using uninoculated control, pathogen-inoculated control and bacterial antagonist-inoculated control *via* GC/MS (GC Clarus 500 Perkin Elmer, USA) with reference to the NIST 2005 MS data library.

### *In silico* molecular docking study of *R. solani*

#### Protein targets of *R. solani* utilized for molecular modeling

A molecular docking study was performed to predict the potential biomolecules that bind with the protein targets of *R. solani*. Four potential protein targets of *R. solani*, namely Polygalacturonase (UniProt ID:L8X539) (Chen et al., 2017), β1,3 glucan synthase (UniProt ID: L8WXK6) (Bhaskar Rao et al., 2020), Pectinesterase (UniProt ID:L8X224) (Prabhukarthikeyan et al., 2022) and Actin like protein ARP6 (UniProt ID: A0A8H3CCY1) (Dallakyan and Olson, 2015), that may have a pathogenesis role were chosen, and the protein sequences were retrieved from the Uniprot database (https://www.uniprot.org/, 2024-05). The sequences were compared with other organisms using the Basic Local Alignment Search Tool (BLAST) in the NCBI database. Based on the similarity of the sequences from the BLAST analysis, templates for homology modeling were selected. Homology modelling was employed for the hypothetical protein structures from the SWISS-MODEL server (https://swissmodel.expassy.org/). The protein targets were validated utilizing the Ramachandra plot of the PROCHECK tool from the Structural Analysis and Verification Server (SAVES v6.1, Meta server) (http://saves.mbi.ucla.edu/) in order to ensure the accuracy of the protein model.

#### Identification of biomolecule ligands for molecular docking

The bioactive compounds produced by the strain B13 alone and its di-trophic interaction with *R. solani* showing high peak areas were specifically chosen as the ligands for docking. The three-dimensional (3D) structures of the ligands were retrieved in SDF format from the PubChem database (https://pubchem.ncbi.nlm.nih.gov/). The commercial fungicide carbendazim was used as a respective reference ligand molecule.

#### Molecular docking and virtual screening

Molecular docking was carried out employing the PyRx 0.8 AutoDock vina module (Singh et al., 2002). Using PyRx software 0.8 version “make macromolecule option”, protein preparation was performed. All ligand structures underwent minimization using a conjugate gradient, involving 200 steps of the first-order derivative optimization process and commercial molecular mechanics parameters Unified Force Field (UFF). The binding site pockets for the targets were identified using the Computed Atlas Topography of Proteins CASTp 3.0 server. During the docking protocol execution, the ligands were permitted to create flexible conformations and orientations with an exhaustiveness value of 8. The interactions of the docked conformations of the protein–ligand complexes were visualized using BIOVIA Discovery studio client 2021 (https://www.3ds.com/products-services/biovia/) for visualization. To distinguish between the receptor, ligand and interacting atoms, different colors were assigned to each of them.

### *In vivo* challenge experiment

An *in vivo* challenge experiment was conducted to test the efficacy of the isolated bacterial strain B13 under glass-house conditions (temperature range of 25–30°C and a relative humidity of 60–90%). The experiment was performed in a completely randomized block (CRD) design, with the three treatments replicated thrice with three plants per replication. The treatments included (1) pathogen inoculation alone (*R. solani*), (2) seed treatment and Foliar spraying with B13 along with a challenge inoculation of *R. solani* (8 × 10^8^ cfu/mL) and (3) healthy control.

Seeds of the rice cultivar Co43 were surface sterilized and raised in an earthen pot. When the plants were 50 days old, a mycelial disc of *R. solani* (diameter 2 mm) was inoculated beneath the rice leaf sheath covered with absorbent cotton (IRRI, 2002). Strain B13 was utilized by spraying after ten days of pathogen inoculation on the rice plants. The disease severity was assessed on the 10th day post-treatment using the 0–9 standard (Yang et al., 2017), and the disease index and control efficacy were calculated with the following formula (Azizi et al., 2017):


$$\text{Disease index}=\frac{\text{Sum of all individual ratings}}{\text{Total no}.\text{ of tillers observed}}\times \frac{100}{\text{Maximum disease grade}}$$

### Study of defense gene expression in rice plant through RT-qPCR

To understand the induction of the resistance response against *R. solani* infection, differential expression of some antifungal defensive genes, namely WRKY 45 (transcription factor), WAK 85 (wall associated kinases), CERK 1 (chitin elicitor receptor kinase 1), LOX (lipoxygenases), PR1 (pathogenesis related protein), JAZ (Jasmonate zim domain protein), FLS 2 (receptor like kinase for flagellin) and PAL (phenylalanine ammonia lyase), of rice susceptible cv. CO43 by the endophyte B13 in the presence of *R. solani*, a glass-house experiment was conducted with four treatments: (1) interaction (spraying of rice plants with B13 and challenge inoculated with *R. solani*), (2) biocontrol (spraying of rice plants with B13 alone), (3) inoculated control (*R. solani* alone) and (4) healthy control (Mock). One hundred milligrams of rice plant tissue were collected separately from all the sets of the *in vivo* experiment and carefully brought to the laboratory for RNA extraction. Total RNA was extracted from the rice plants treated with *B. velezensis* challenged with *R. solani* using Trizol (Sigma Aldrich, St. Louis, MO, USA) at 0 h, 24 h, 48 h, 72 h and 96 h post-inoculation with *R. solani.* Likewise, RNA was extracted from untreated healthy control, plants treated with *B. velezensis* B13 alone and *R. solani*-inoculated control (Livak and Schmittgen, 2001). The concentration and purity of total RNA were quantified using a NanoDrop ND1000 spectrophotometer.

RNA was converted to cDNA utilizing the Thermo Fischer Scientific-Revert Aid First Strand cDNA Synthesis Kit (cat. #RR820A). An optimal nucleic acid quality was indicated by a ratio of 1.8 + 0.2. Subsequently, the cDNA was diluted 10-fold and utilized for qRT-PCR analysis, which was carried out in a BIO RAD CFX manager system. For qRT-PCR, the reaction mixture included 1.4 µL of cDNA template, 5 µL of SYBR Green master mix (KAPA SYBR@FAST for Light Cycler 480), 0.8 µL of 10 µM forward primer and 0.8 µL of 10 µM reverse primer. Using nuclease-free water, the final volume was adjusted to 10 µL. The PCR program included denaturation at 95°C for 1 min, followed by 50 cycles of amplification at 95°C for 10 s, 60°C for 30 s and 72°C for 30 s. Subsequently, a standard melting temperature analysis was performed. Actin was used as a housekeeping gene to normalize the gene expression. For each defense gene expression analysis, three biological replicates and two technical replicates were consistently maintained.

### Statistical analysis

Fold changes in gene expression were determined utilizing the formula ΔΔCt = ΔCt sample–ΔCt reference. The relative fold changes in transcript levels were graphically represented by converting the ΔΔCt value to 2^-ΔΔCt^ (Khaskheli et al., 2020).

## Results

### Isolation and screening of endophytic bacteria

**Figure 1:**
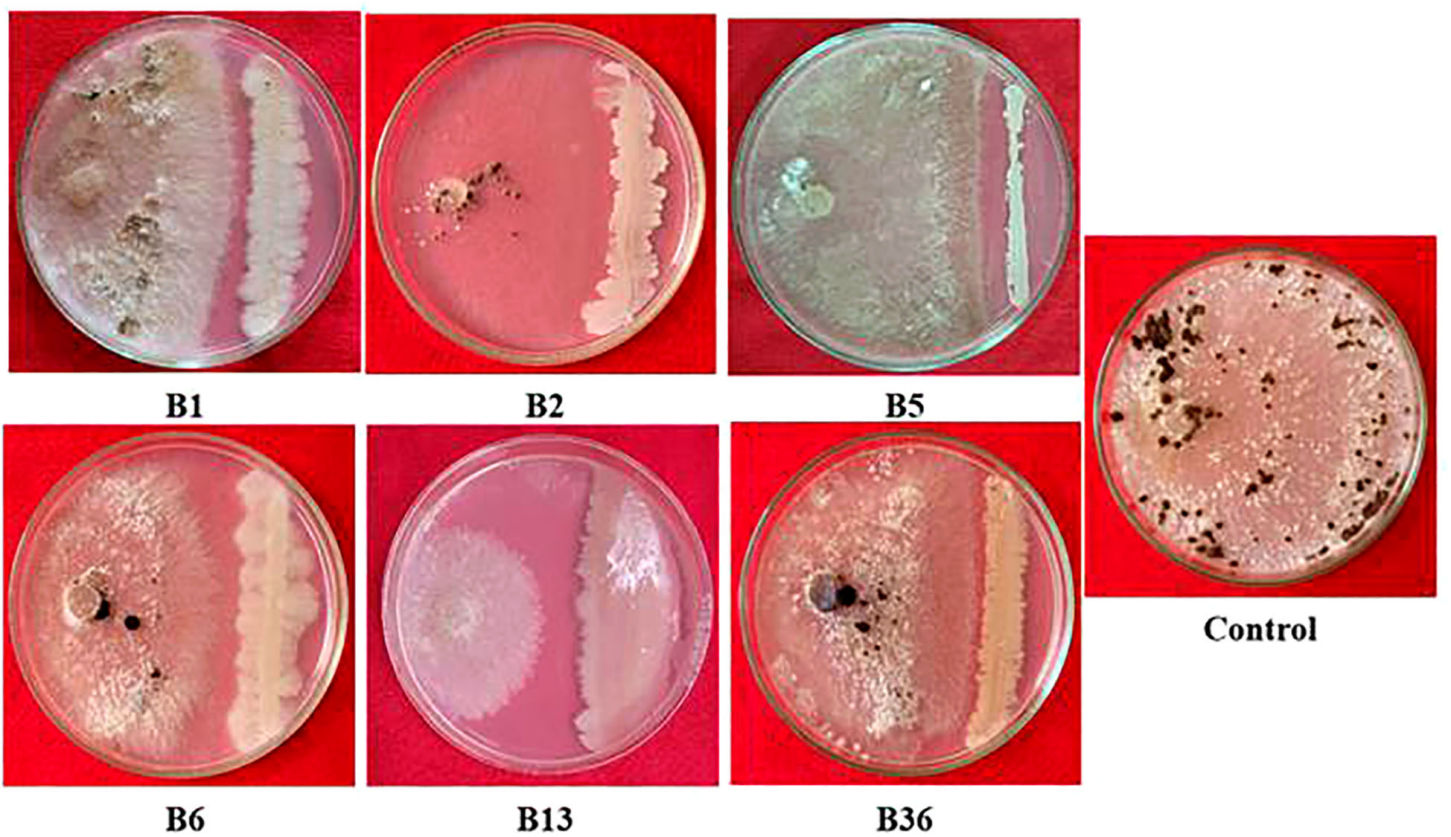
Antagonistic potential of effective rice bacterial endophytes against *R. solani*.

A total of forty endophytic bacteria were isolated and evaluated for their antagonistic activity against *R. solani* in a dual-culture assay (**Supplementary Figure S1**). Among them, six endophytic bacterial isolates showed antagonism against *R. solani*. The results showed that strain B13 displayed the highest inhibitory and antagonistic effects against the pathogen with the highest percentage of mycelia inhibition (77.33 (± 1.27) %) (**Table 2**). Therefore, based on the results obtained in the screening, isolate B13 was utilized for further study (**Figure 1**).

**Table 2:** Antagonistic activity of effective endophytic bacteria against *R. solani* by dual-culture method.

Isolate Number	Average Diameter of Fungal Colony (cm)	Percent Inhibition over Control *
B1	3.93 (± 0.15)	56.23 (± 1.68)
B2	3.77 (± 0.15)	58.10 (± 1.73)
B5	4.40 (± 0.10)	51.10 (± 1.10)
B6	2.30 (± 0.10)	74.40 (± 1.10)
B13	2.03 (± 0.12)	77.33 (± 1.27)
B36	2.87 (± 0.15)	68.10 (± 1.73)
Control	9.00	–
C.D.	0.21	2.39
SE(m)	0.07	0.78

*The statistical analysis was performed using one-way analysis of variance and Duncan’s multiple range test (*p<* 0.05). Values in brackets are mean ± standard errors of three independent experiments.

### Molecular identification

Upon 16S rRNA gene sequencing, strain B13 was identified as *B. velezensis*. The 1500 bp sequence of the strain B13 was submitted to the NCBI database and provided with the accession number OQ941779.1. It showed 99.63% homology from the NCBI database with *B. velezensis* strain R-71003 (ON358418.1). A neighbor joining phylogenetic tree was constructed based on the 16S rRNA, which revealed that the isolate B13 formed a cluster with the *B. velezensis* group (**Supplementary Figure S2**).

### Scanning electron microscopy

**Figure 2:**
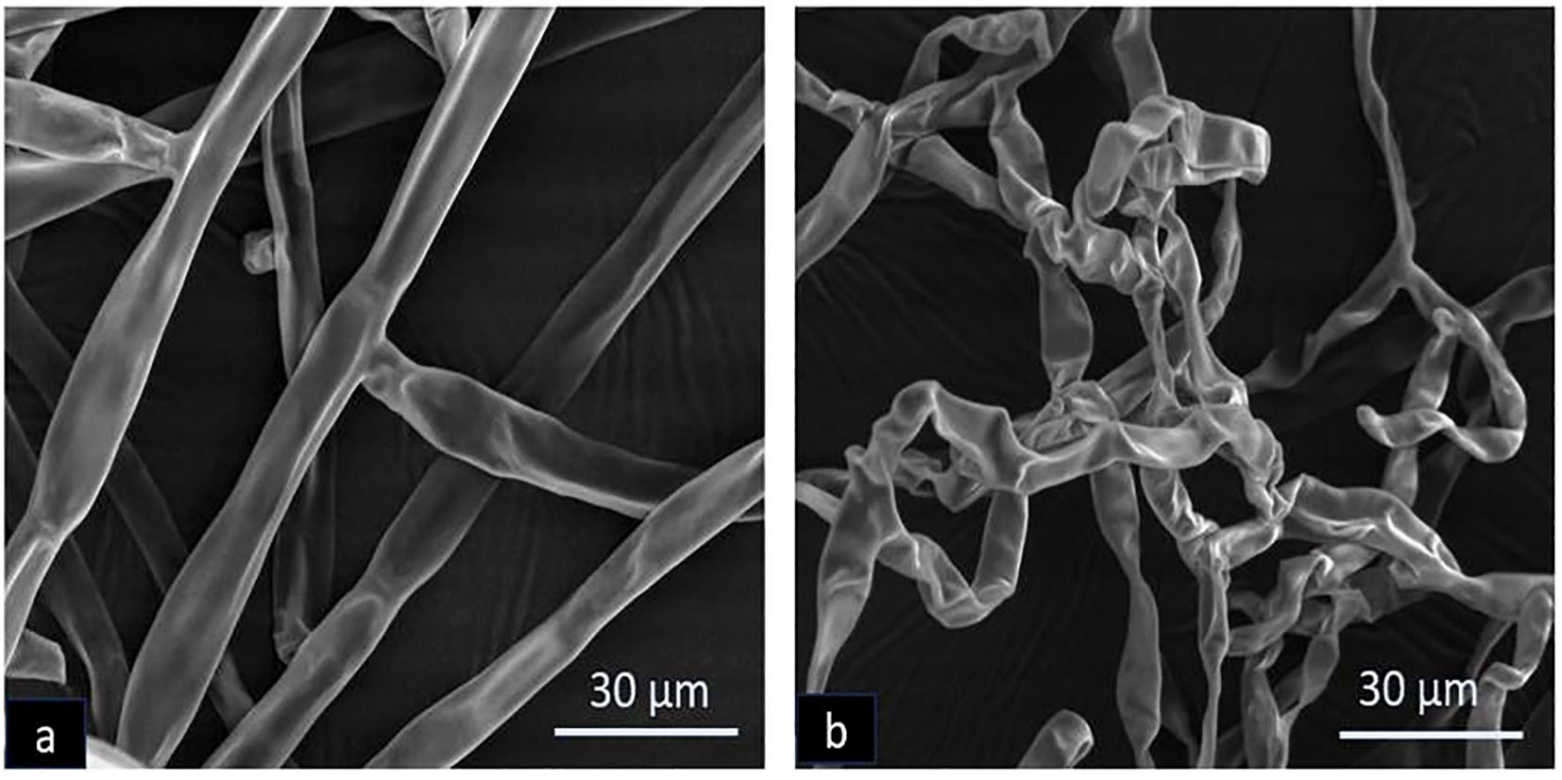
Scanning electron microscopy of morphological deformation of *R. solani* caused by *B. velezensis* B13: **(a)** untreated control and **(b)** treated with B13.

The morphological alterations in the mycelia of *R. solani* caused by volatile compounds from *B. velezensis* B13 were analyzed using scanning electron microscopy. The untreated mycelia were compared with the mycelia inoculated with B13. The results showed that the untreated mycelium exhibited a smooth and structurally intact appearance. Conversely, the mycelium of *R. solani* treated with B13 displayed a distorted and deformed morphology with an uneven thickness (**Figure 2**). Based on the results of the observed hyphal malformation under SEM, strain B13 had a significant impact on the mycelium of *R. solani*.

### GC/MS analysis of secondary metabolites produced by *B. velezensis* B13, *R. solani* and their di-trophic interaction

**Figure 3:**
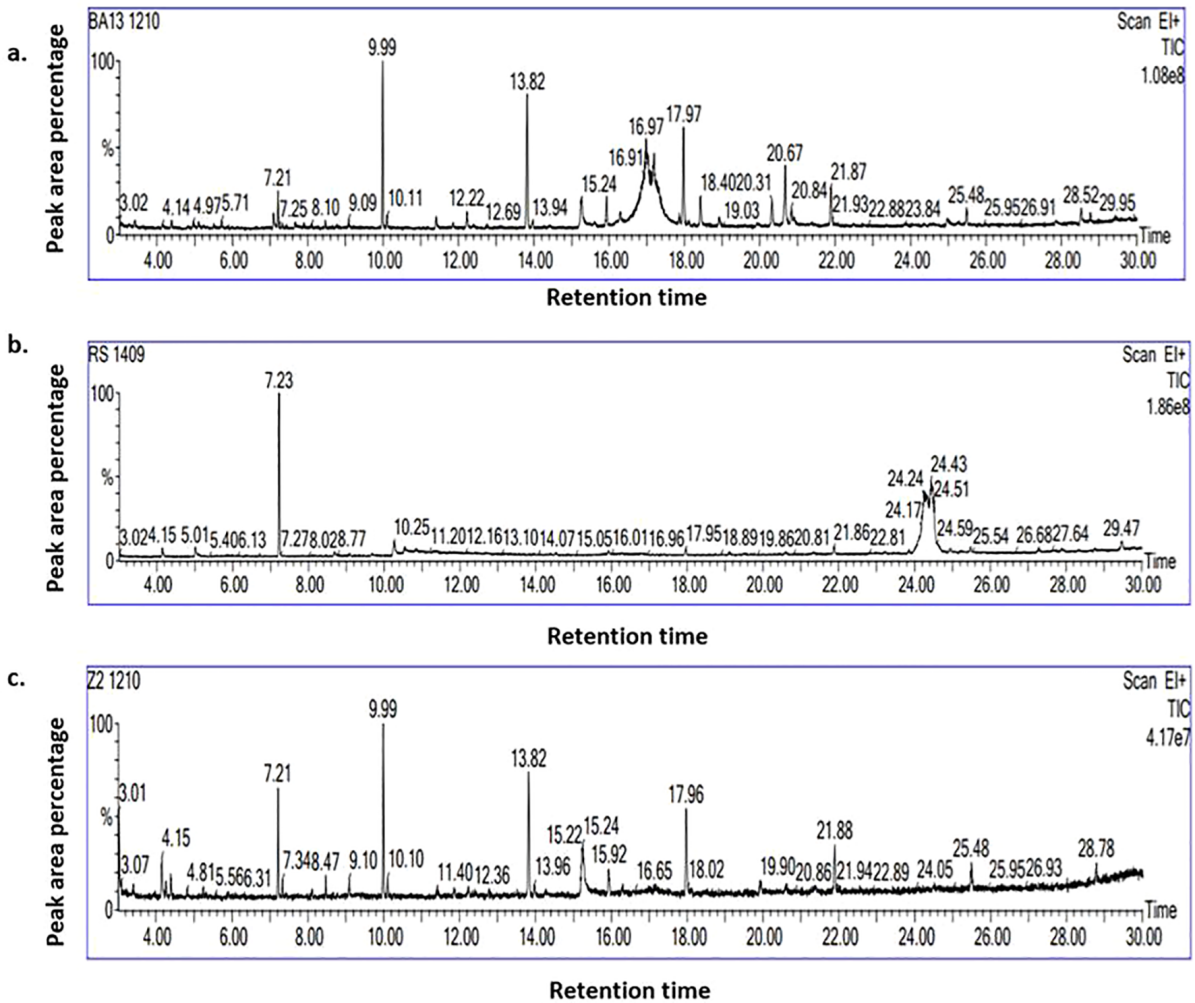
GC–MS chromatogram of biomolecules produced by **(a)** *B. velezensis* B13; **(b)** *R. solani* and **(c)** during the interaction of *B. velezensis* B13 and *R. solani*.

*B. velezensis* B13, *R. solani* and their di-trophic interaction were profiled for a total of 39 biomolecules upon elimination of compounds in the PDA medium (control). A total of twelve bioactive metabolites produced by *R. solani* in PDA medium were identified: Naphthalene, squalene, Trans-geranylgeranio, Oleic acid, Hexadecanoic acid 1 4- methyl ester, 1-Hexadecanol 2-methyl, Phenol, Tetradecanoic acid, 1 2 -methyl methyl ester, Dichloroacetic acid, Trichloroacetic acid tridecyl ester and 1-Hexadecane (**Supplementary Table S1**; **Figure 3a**).

In the absence of *R. solani*, *B. velezensis* B13 generated a total of 17 compounds, 14 of which were unique to *B. velezensis* B13, which included Bis(2-ethylhexyl) phthalate, Octahydro-2H-pyrido(1,2-a) pyrimidin-2-one, 1-Undecanol, 4a(2H)-Naphthalenol octahydro-trans, Hexadecen-1-ol trans-9, Furan 2-ethenyl, Phthalic acid di (6-methylhept-2-yl) ester, Octadecanoic acid 2-propenyl ester, Dodecyl acrylate, Formamide N-(4,6-diamino-5-pyrimidinyl), Ergotamine, 9-Eicosene (E), Phenol 3,5-bis(1,1-dimethylethyl) and Di-Aspartic acid (**Supplementary Table S2**; **Figure 3b**).

During the interaction of *B. velezensis* B13 with *R. solani*, 14 biomolecules were identified, among which 12 molecules were profiled during interaction: Benzoic acid 3-amino-6-(1-pyrrolidinyl), Methyl 5,9-heptadecadienoate, 1-Octene 3,7-dimethyl, 5,8-Dimethoxyquinoxaline, Cyclotetradecane, Formic acid phenyl ester, 3′,8,8′-Trimethoxy-3-piperidyl-2,2′-binaphthalene-1,1′,4,4′-tetrone,4-(2,4,4-Trimethyl-cyclohexa-1,5-dienyl)-but-3-en-2-one, 2-Propenoic acid, butyl ester, 1-Docosene, Octadecane 1-chloro- and 1-Dodecane (**Supplementary Table S3**; **Figure 3c**). None of the biomolecules were produced in common by *R. solani* and its interaction with *B. velezensis* B13. The Venn diagram of the differentially expressed bioactive metabolites revealed phenol as a common compound during the interaction of the endophytic bacteria *B. velezensis* B13 with *R. solani*, *B. velezensis* B13 alone and *R. solani* alone (**Supplementary Figure S3**).

### Molecular modeling and validation of protein targets

The protein targets, like Actin like protein ARP6, β1,3 glucan synthase, Pectinesterase and Polygalacturonase, were chosen as receptors due to their significant physiological functions (**Supplementary Table S4**). The three-dimensional structures of the proteins were predicted using the SWISS-MODEL for molecular docking studies. The sequence similarity between the template and 3D-modelled structure of the proteins was validated through the Ramachandran plot (**Supplementary Table S5**). The Ramachandran plot analysis of the 3D structure of modeled Actin like protein ARP6, β-1,3 glucan synthase, Pectinesterase and Polygalacturonase revealed 90.5%, 92.3%, 91% and 88.5% of amino acid residues in the most favored region, respectively (**Supplementary Figure S4**).

### Molecular docking and virtual screening

**Figure 4:**
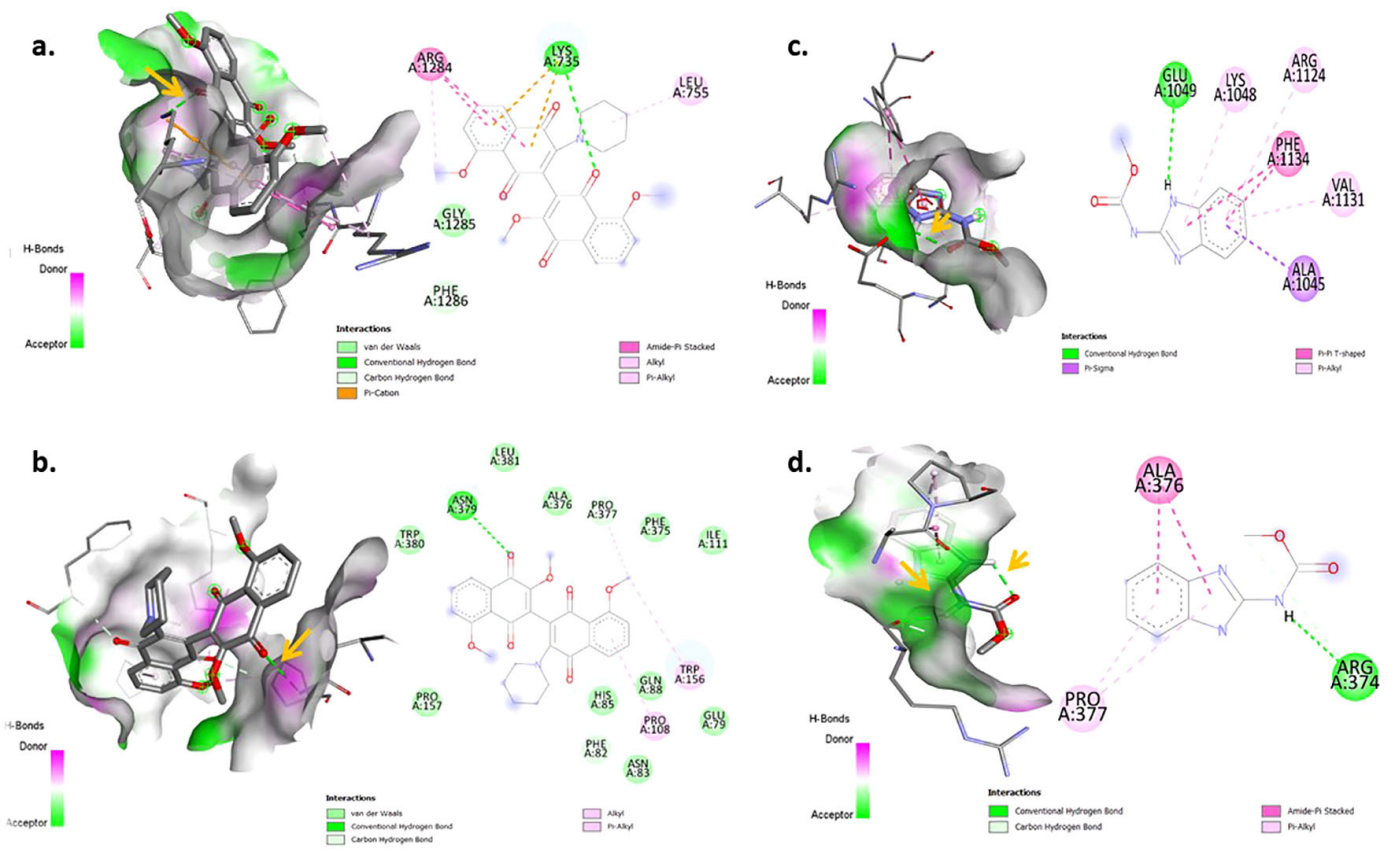
Visualization of Molecular docking interaction in 3D (left) and 2D (right): **(a, b)** Docked complex of 3′,8,8′-Trimethoxy-3-piperidyl-2,2′-binaphthalene- 1,1′,4,4′-tetron with active site residues of β-1,3 glucan synthase and Actin like protein ARP 6 and x **(c, d)** Docked complex of carbendazim with active site residues of β-1,3 glucan synthase and Actin like protein ARP6. Arrow represents the conventional hydrogen bond.

Four protein targets of *R. solani* were docked with 12 compounds and exhibited different binding affinity towards the 12 compounds. The results showed that, out of 12 compounds, 3′,8,8′-Trimethoxy-3-piperidyl-2,2′-binaphthalene-1,1′,4,4′-tetrone interacted well with all four protein targets and displayed a higher binding affinity than the reference ligand carbendazim. The compound had a binding affinity value of −7.6 kcal/mol with the target Actin like protein ARP6 (H bonds: ASN 379), −7.7 kcal/mol with the target β-1,3 glucan synthase (H bonds: LYS 735), −7 kcal/mol with the target Pectinesterase (H bonds: GLN 76, ARG 152) and −6.5 kcal/mol with the target Polygalacturonase (H bonds: LYS 242) (**Figure 4a**). Similarly, compound 4-(2,4,4-Trimethyl-cyclohexa-1,5-dienyl)-but-3-en-2-one showed a good interaction of −6.9 kcal/mol for β-1,3 glucan synthase (H bonds: ASN 1096), −6.1 kcal/mol for Actin like protein ARP6 (H bonds: LYS 174, THR 188) and −5.7 kcal/mol for Pectinesterase (H bonds: ILE 219) (**Figure 4b**). Compound Hexadecen-1-ol, trans-9 showed a good interaction of −5.2 kcal/mol with Actin like protein ARP6 (H bonds: SER 202). The compound Phthalic acid, di (6-methylhept-2-yl) ester showed a good binding energy of −6.2 kcal/mol for Actin like protein ARP6 (H bonds: ASN 379) and −5.4 kcal/mol for pectinerase (H bonds: GLN 54, GLN 76). The binding energy for the control carbendazim was −5.0 kcal/mol for Actin like protein ARP6 (H bonds: ARG 374), −6.8 kcal/mol for β-1,3 glucan synthase (H bonds: GLU 1049), −5.5 kcal/mol for polygalacturonase (H bonds: TYR 251) and −5.3 kcal/mol for pectinesterase (H bonds: GLY 111, THR 141, SER 112, THR 113) (**Table 3**; **Figures 4c, d**).

**Table 3:** Molecular docking for the interaction of metabolites of *B. velezensis* B13 with the protein targets of *R. solani*.

S. No	Compound Name	Binding Affinity (Kcal/mol) of Biomolecules on Different Targets				Interacting Amino Acids (H Bonds Formed)			
		Actin Like Protein	β-1,3 Glucan Synthase	Pectin Esterase	Poly Galacturonase	Actin Like Protein	β-1,3 Glucan Synthase	Pectin Esterase	Poly Galacturonase
1	4-(2,4,4-Trimethyl-cyclohexa-1,5-dienyl)-but-3-en-2-one	−6.1	−6.9	−5.7	−4.8	LYS 174, THR 188	ASN 1096	ILE 219	GLY 33
2	Hexadecen-1-ol, trans-9	−5.2	−5.4	−4.2	−4	SER 202	PHE 1602	GLN 61	GLN 43
3	3′,8,8′-Trimethoxy-3-piperidyl-2,2′-binaphthalene-1,1′,4,4′-tetron	−7.6	−7.7	−7	−6.5	ASN 379	LYS 735	GLN 76, ARG 152	LYS 242
4	Phthalic acid, di (6-methylhept-2-yl) ester	−6.2	−6.6	−5.4	−5.1	ASN 379	ASN 409, ASN 412	GLN 54, GLN 76	LYS 217, TYR 251, SER 190
5	Octahydro-2H-pyrido(1,2-a) pyrimidin-2-one	−4.9	−5.4	−4.5	−4.5	CYS 18	ALA 1224	ASP 98, ARG 152	SER 190, LYS 217
6	1-Undecanol	−4.9	−4.8	−3.9	−3.7	HIS 14	VAL1172, ALA 1171	SER 177, TYR 121	ASP 163
7	4a(2H)-Naphthalenol, octahydro-, trans	−4.9	−6.3	−5	−4.7	GLN 88	ALA 1045	GLN 54	GLN 161, HIS 184
8	1-Hexadecanol	−4.0	−4.2	−3.9	−3.1	GLN 88	GLU 1419	ASP 82	ALA 136
9	2-Propenoic acid, butyl ester	−4.1	−4.4	−4.1	−3.9	ARG 106, GLN 88	ARG 1030, GLN 119, GLN 1050	GLN 76	ARG 215, THR 254
10	Furan, 2-ethenyl-	−3.9	−5.4	−4.2	−4.3	ARG 255	THR 465	TRP 154	THR 254
11	1-Octene, 3,7-dimethyl-	−4.8	−4.8	−4.6	−4.4	ASN 83	GLU 747, PHE 1286	ASN 22, ASP 77	GLN 161
12	5,8-Dimethoxyquinoxaline	−5.2	−6.1	−4.8	−4.3	GLN 88	THR 1288	GLN 76, TRP 154	SER 106, SER 147
13	Carbendazim (Positive control)	−5.0	−6.8	−5.3	−5.5	ARG 374	GLU 1049	GLY 111, THR 141, SER 112, THR 113	TYR 251

### *In vivo* evaluation of *B. velezensis* B13 against *R. solani* infected rice plants

**Figure 5:**
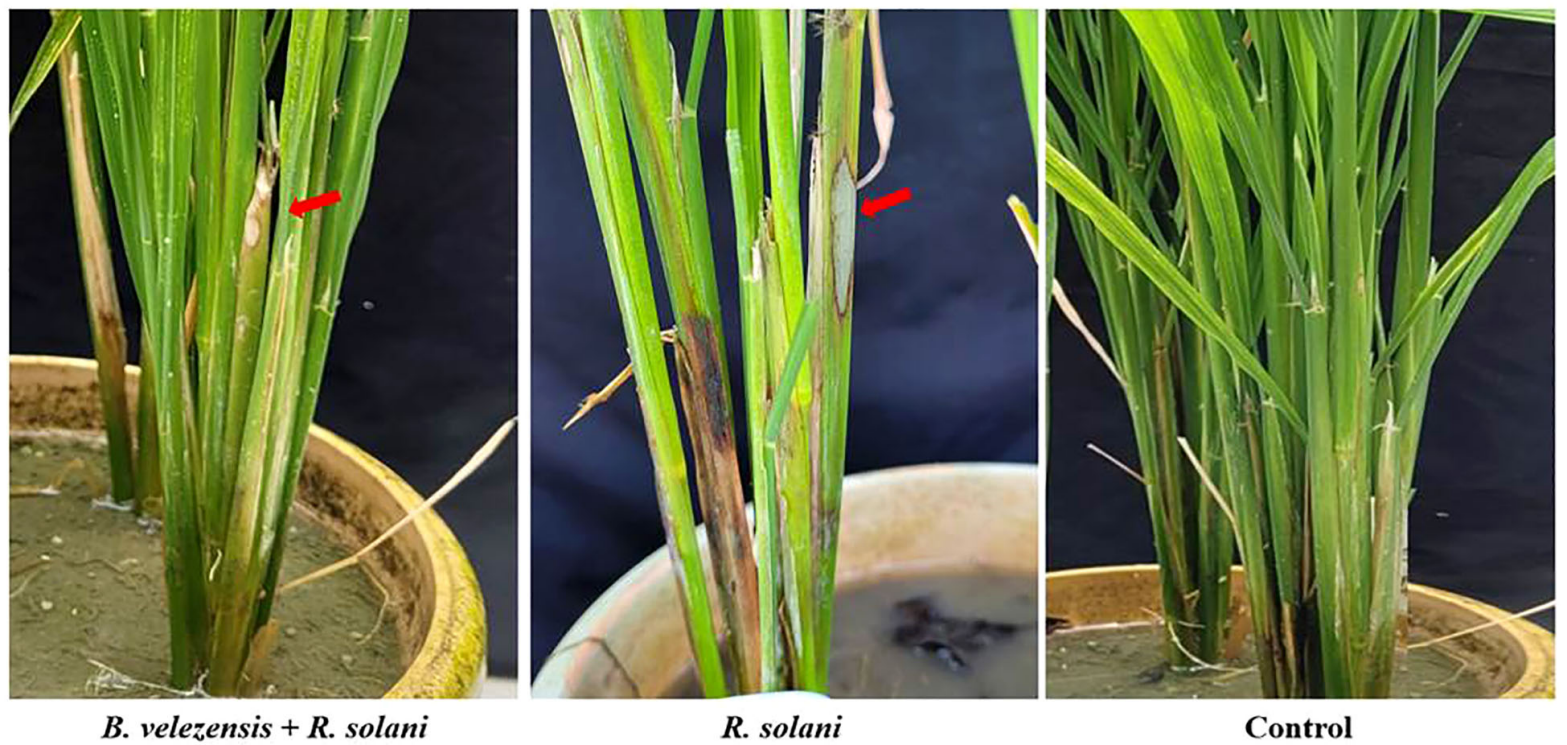
*In vivo* antagonistic activity of *B. velezensis* B13 against *R. solani* infection of rice. (Arrows indicate the development of lesions upon inoculation of *R. solani*).

The antagonistic nature of *B. velezensis* B13 was studied under glass-house conditions for determining its effect in suppressing sheath blight disease caused by *R. solani*. The rice plants inoculated with *R. solani* showed a disease index of 65.46 ± 1.12, while application of *B. velezensis* B13 resulted in a reduced disease index of 54.43 ± 0.03 (**Table 4**). Both seed treatment and foliar spraying of *B. velezensis* B13 resulted in a disease reduction of 16.8 ± 0.2 compared to the control (**Figure 5**).

**Table 4:** *In Vivo* antagonistic effect of *B. velezensis* B13 against rice sheath blight under glass-house conditions.

Treatments	Plant Height (cm)	Percent Disease Index	Percent Reduction over Control	Grain Yield/Plant (g)
Inoculated with *R. solani* + (Seed treatment + Foliar spraying with *B. velezensis*)	74.96 ± 0.50	54.43 ± 0.03	16.8 ± 0.2	23.46 ± 0.44
Inoculated with *R. solani*	74.13 ± 0.95	65.46 ± 1.12	0	19.33 ± 0.38
Healthy control	76.13 ± 1.16	0	0	20.93 ± 0.29

Values are the mean of three replications ± standard error (n = 10).

### Induction of plant defense gene transcripts by endophytic bacterium

The rice plants treated with *B. velezensis* B13 challenged with or without *R. solani* altered the expression of transcription factor (WRKY45), wall associated kinases 85 (WAK 85), chitin elicitor receptor kinase 1 (CERK1), lipoxygenases (LOX), jazmonate zim domain protein (JAZ), receptor like kinase for flagellin (FLS2), phenylalanine ammonia lyase (PAL) and pathogenesis related protein 1 (PR1) genes responsible for plant defense.

**Figure 6:**
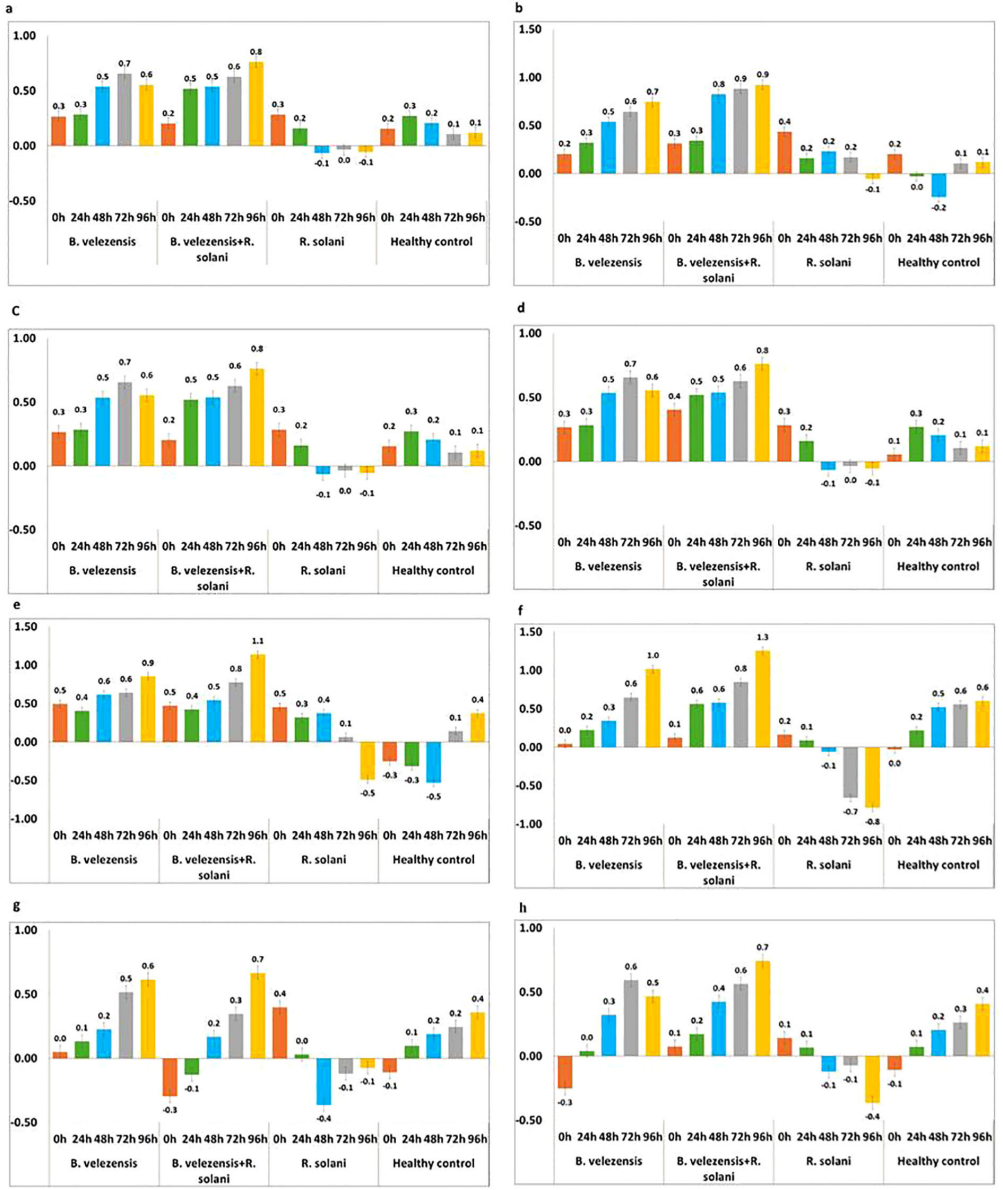
Expression pattern of defense genes in rice plants treated with *B. velezensis* B13 under mono-, di- and tri-trophic interactions at different time intervals. **(a)** Expression pattern of WRKY; **(b)** Expression pattern of JAZ; **(c)** Expression pattern of WAK; **(d)** Expression pattern of PR1; **(e)** Expression pattern of PAL; **(f)** Expression pattern of LOX; **(g)** Expression pattern of CERK1; **(h)** Expression pattern of FLS2.

The expression of WRKY transcript was upregulated in all the treatments from 0 to 24 h. However, upregulation was more significant in the plants treated with *B. velezensis* B13 challenged with *R. solani* in all the intervals, and a 0.8-fold increase in WRKY 45 transcript was observed at 96 h. In addition, the plants treated with *B. velezensis* showed an increase in expression of WRKY transcript up to 0.7-fold after 72 h, which was reduced to 0.1-fold after 72 h. The transcript level in the *R. solani* inoculated control declined after 48 h. Furthermore, the expression level of WRKY transcript in the untreated healthy control increased up to 0.3-fold and declined after 24 h (**Figure 6a**).

JAZ gene was a key regulator in the plant defense response through the JA signaling cascade. Upregulation of JAZ was noticed at 0 h in all the treated and untreated plants. The level of JAZ was more pronounced in the plants treated with *B. velezensis* B13 challenged with *R. solani* after 24 h, which showed a 0.9-fold increase after 72 to 96 h. The expression level of JAZ was upregulated in the *B. velezensis*-treated plants, which showed a 0.7-fold increase after 72 h. The plants inoculated with *R. solani* showed an upregulation from 0 to 72 h and displayed downregulation after 72 h (**Figure 6b**).

Assessing the expression of WAK transcript revealed a significant increase in the plants treated with *B. velezensis* challenged with *R. solani*, which showed up to a two-fold increase after 72 h. Similarly, the plants treated with *B. velezensis* also showed an increase in WAK transcript from 0 h to 96 h, which showed up to a 1.9-fold increase after 72 h. The expression of WAK transcript was downregulated after 24 h in the plants inoculated with *R. solani*, while in the healthy control, there was an upregulation from 0 h to 96 h (**Figure 6c**).

PR1 gene is regarded as a key regulator gene for systemic acquired resistance. The expression of PR1 was upregulated from 0 to 24 h in all the treatments, and a characteristic downregulation of the PR1 transcript was noticed in the *R. solani*-inoculated plants after a 24 h interval. The significant upregulation of PR1 was observed in the plants treated with *B. velezensis* challenged with *R. solani*, which increased the transcript level to 0.8-fold after 72 h. The plants treated with *B. velezensis* showed an increase in the expression level from 0.3 to 0.7-fold in 0 to 72 h and showed a decline to 0.1-fold after 72 h. The expression of PR1 was upregulated from 0 h to 96 h in the untreated control plants (**Figure 6d**).

The expression level of PAL was upregulated in both the plants treated with *B. velezensis* alone and plants treated with *B. velezensis* challenged with *R. solani*. The level of induction of PAL was increased to 1.1-fold after 72 h in the plants treated with *B. velezensis* challenged with *R. solani*. A comparison of the expression of PAL in the *B. velezensis*-treated plants reflected a 0.9-fold increase after 72 h, while the pathogen-inoculated control showed an upregulation of PAL from 0 h to 72 h and a downregulation after 72 h. The PAL expression level in the untreated healthy control showed a downregulation from 0 h to 48 h and an upregulation after 48 h (**Figure 6e**).

The upregulation of LOX was noticed in the plants treated with *B. velezensis* and also in the plants treated with *B. velezensis* co-challenged with *R. solani*. The level of induction of LOX was increased to 1.3-fold after 72 h in the plants treated with *B. velezensis* challenged with *R. solani*. A comparison of the expression of LOX in the *B. velezensis*-treated plants reflected a one-fold increase after 72 h, while in the pathogen-inoculated control, the expression of LOX was downregulated after 24 h. The untreated plants showed an upregulation after 24 h, and a 0.6-fold increase was noticed from 72 h to 96 h (**Figure 6f**).

The expression level of CERK transcript varied between the different treatments. The expression of the CERK gene was upregulated in the *B. velezensis*-treated plants, and an increase of 0.6-fold was observed after 72 h. The rice plants treated with *B. velezensis* B13 challenged with *R. solani* showed upregulation after 24 h, and an increase of up to 0.7-fold was noticed after 72 h, which declined to 0.3-fold after 72 h. The activity of CERK in the *R. solani*-inoculated control was significantly downregulated after 24 h to 96 h. The untreated control plants showed an upregulation of the CERK gene after 24 h and an increase to 0.4-fold after 72 h (**Figure 6g**).

The transcription rate of the FLS2 gene was upregulated in the *B. velezensis*-treated plants after 24 h and showed a 0.6-fold increase after 48 h and a decrease to 0.1-fold after 72 h. The plants treated with *B. velezensis* challenge inoculated with *R. solani* showed an increase in FLS2 from 0.1-fold to 0.7-fold in 0 h to 96 h. The plants inoculated with *R. solani* showed downregulation of FLS after 24 h intervals. The untreated healthy control showed an upregulation of the FLS2 gene after 24 h and an increase to 0.4-fold after 72 h (**Figure 6h**).

## Discussion

Biological control is considered a sustainable strategy for the management of plant diseases by reducing the need for harmful pesticides and, thereby, promoting a healthier ecosystem. Recently, the endophytic bacteria suppression of plant diseases has received much attention (Kumar et al., 2020). The diversity of the different endophytic bacteria from the rice plant that are antagonistic to *R. solani*, which causes sheath blight in rice, is well established (Jeong et al., 2017; Khaskheli et al., 2020). Among the different endophytic bacteria, *Bacillus* species quench the plant pathogens by producing antifungal biomolecules and antimicrobial peptides and by inducing an immune response (Aloo et al., 2019; Nakkeeran et al., 2019). Because of their wide range of bioactive substances, the *Bacillus* species were considered a more effective and eco-friendlier supplement when it comes to suppressing soil-borne diseases (Sun et al., 2017). They are also a source of secondary metabolites of biotechnological interest with pharmaceutical applications (Balderas-Ruíz et al., 2020).

In our current investigation, approximately forty endophytic bacteria were isolated from the rice plants. Among them, six isolates showed significant mycelial growth suppression against *R. solani.* The strain B13 showed the highest inhibition activity both under *in vitro* and *in vivo* conditions. The strain B13 was identified as *Bacillus velezensis* based on 16S rRNA gene sequencing. The potentiality of *B. velezensis* has been extensively studied as a biological control agent for controlling numerous fungal plant pathogens (Saravanan et al., 2022; Soliman et al., 2022). *B. velezensis* NKG-2 was able to produce hydrolytic enzymes associated with the breakdown of the fungal cell wall and was effective in suppressing phytopathogens (Myo et al., 2019). The interaction of antifungal biomolecules produced during the di-trophic interaction with *R. solani* through scanning electron microscopy revealed distorted and deformed mycelium with an uneven thickness. Similar ultrastructural changes in *R. solani* were observed during its di-trophic interaction with *B. safensis* Y246 (Zhu et al., 2023).

*Bacillus velezensis* harbors numerous biosynthetic gene clusters with the potential to produce a wide variety of metabolites (Su et al., 2024). The secondary metabolites produced by *B. velezensis* and their di-trophic interaction with *R. solani* were analyzed using GC–MS chromatography. Our study identified a diverse range of bioactive secondary metabolites of *B. velezensis* B13, which exhibit strong antifungal activity, effectively inhibiting the mycelial growth of *R. solani.* The antifungal property of these bacteria may be related to several chemical classes, including esters, alcohols, fatty acids, aldehydes, alkaloids, tertiary amines and ketones. Similarly, co-culturing *B. velezensis* B13 with *R. solani* stimulated the production of secondary metabolites, *viz*., Benzoic acid 3-amino-6-(1-pyrrolidinyl), Methyl 5,9-heptadecadienoate, 1-Octene 3,7-dimethyl, 5,8-Dimethoxyquinoxaline, Cyclotetradecane, Formic acid phenyl ester, 3′,8,8′-Trimethoxy-3-piperidyl-2,2′-binaphthalene-1,1′,4,4′-tetrone,4-(2,4,4-Trimethyl-cyclohexa-1,5-dienyl)-but-3-en-2-one, 2-Propenoic acid, Cetyl alcohol, Isobutyl acrylate and 1-Dodecane. The biosynthetic pathway of these compounds involves non-ribosomal peptide synthetases (NRPS) and polyketide synthases (PKS), enabling the production of antimicrobial lipopeptides (e.g., surfactin, fengycin, bacillomycin D and bacillibactin) and polyketides (e.g., macrolactin, difficidin, and bacillaene) (Cawoy et al., 2015; Rabbee et al., 2019). Various secondary metabolites produced by the EA fraction of *B. velezensis* Lle-9, *viz*., cyclopeptides, linear peptides and some antibiotics were reported which possessed antifungal properties (Khan et al., 2020).

In this study, molecular docking was performed to identify the potential of 12 secondary metabolites produced by *B. velezensis* against the four targeted proteins (Actin like protein ARP6, Polygalacturonase, β1,3 glucan synthase and Pectinesterase) of *R. solani.* All the metabolites were found to interact with the target proteins. Among those, 3′,8,8′-Trimethoxy-3-piperidyl-2,2′-binaphthalene-1,1′,4,4′-tetrone exhibited the highest binding energy with all the protein targets. Several studies carried out on the interaction between protein and ligand provided valuable insights into the mechanisms by which these compounds combat pathogens (Malik et al., 2019; Gorai et al., 2023; Yasmin et al., 2023). The compound 3′,8,8′-trimethoxy-3-piperidyl-2,2′-binaphthalene-1,1′,4,4′-tetrone derived from the *B. cereus* strain KSAS17 exhibited antifungal properties against phytopathogenic fungus, *Sclerotium bataticola* (Al-Askar et al., 2024). It also shows broad-spectrum potential in antimicrobial, immunomodulatory and anti-inflammatory activities (Amer et al., 2019). Phthalic acid, a dicarboxylic compound derived from benzoic acid, is recognized for its antifungal properties. Its ester derivatives demonstrate insecticidal, antibacterial, and allelopathic effects, indicating their potential to enhance the resilience of plants, algae, and microorganisms against both biotic and abiotic stresses, thereby supporting their competitive survival (Awan et al., 2023). The antifungal activity of the Phthalic acid, di (6-methylhept-2-yl) ester produced by *B. amyloliquefaciens* against *B. cinerea* was reported by (Nakkeeran et al., 2020). Compound 4-(2,4,4-Trimethyl-cyclohexa-1,5-dienyl)-but-3-en-2-one is a naturally occurring terpenoid ketone which exhibits antimicrobial activity, potentially by disrupting bacterial cell membranes, leading to increased permeability and cell lysis (Idan et al., 2015). Volatile compound hexadecen-1-ol, trans-9 produced by *Serratia plymuthica* affected the mycelial growth of the soil-borne fungus *R. solani* (Kai et al., 2007).

*Bacillus* spp. protects the plants against phytopathogens by triggering systemic resistance, which subsequently results in the upregulation of defense-linked genes (Sahu et al., 2019). Further, bacterial endophytes stimulate the immune system of plants by releasing macromolecules and MAMP molecules in response to host signals (Wei et al., 1991). Our study demonstrated that the rice plants treated with bacterial endophyte *B. velezensis* B13 showed a significant upregulation of defense genes WRKY45 transcription factor, WAK 85, CERK1, LOX, JAZ, FLS 2, PAL and PR1 than in the inoculated control and healthy control plants. Transcription factors play a vital role in triggering plant defense mechanisms by controlling a wide array of signal transduction pathways responsible for carrying out diverse functions. A group of transcription regulators called WRKY can attach to the box in specific promoter regions in target genes and regulate transcription (Eulgem et al., 2000). Proteins of the WRKY family are important regulators of pathogen-associated molecular pattern-triggered immunity (PTI) and effector-triggered immunity (Zhang and Wang, 2005; Chen et al., 2019). WRKY 45 transcription factor in the rice plants treated with *B. velezensis* B13 increased by multiple folds, which might have triggered the constitutive defense response against *R. solani*. Plant cell wall-associated kinases (WAKs) are receptor-like kinases present in multiple plant species possessing an extracellular domain and an intercellular domain that span the plasma membrane (Yang et al., 2019). They play a role in monitoring cell wall integrity and are implicated in pathogen responses (Rui and Dinneny, 2020). Several studies have shown that increased PAL activity, which is a crucial enzyme in the phenylpropanoid pathway of higher plants, is primarily associated with the ability to resist pathogens (Tonnessen et al., 2015). PR1 proteins were the first pathogenesis-related proteins identified in the PR family, associated with plant resistance to pathogens (Showmy and Yusuf, 2020). Tomato plants treated with *B. subtilis* CBR05 systemically induced the enzyme activities of PAL and β-1,3-glucanase, and significant upregulation was observed in PAL transcript of 0.49-fold expression at 72 h post-inoculation (hpi) as compared to its expression at 12 h post-inoculation (hpi) (Chandrasekaran and Chun, 2016). As a cell surface receptor, CERK 1 is essential for triggering innate immunity in response to biotic and abiotic stresses (Shinya et al., 2014). In rice, Chitin Elicitor Receptor Kinase 1 (CERK1) recognizes several elicitor compounds that have similar elicitor motifs, such as chitin as well as peptidoglycan and its derivatives. CERK1 is essential for chitin-mediated signaling and plays a crucial role in fungal resistance. Notably, pathogen effector proteins may interfere with CERK1 function, thereby evading CERK1-mediated recognition. Plants lacking functional CERK1 are not only unresponsive to chitin treatment but also exhibit increased susceptibility to fungal pathogens (Zhang and Zhou, 2010). These multifunctional receptors are essential for plant defense against pathogen invasion (Liu et al., 2012). LOX mediates jasmonic acid (JA) biosynthesis, which is essential for plant development and resists biotic and abiotic stresses (Singh et al., 2022). The rice ortholog receptor-like kinase FLAGELLIN-SENSITIVE 2 (FLS2) can identify the flagellar peptide flg22 and activate a plant immune response (Li et al., 2014). Ultimately, the present investigation highlighted the potential of endophytic *B. velezensis* in preventing pathogenic infections by triggering the WRKY 45 transcription factor and activating defense genes, such as WAK, PAL, LOX, PR1, JAZ, FLS2 and CERK1.

## Conclusion

The present study highlights the potential of *Bacillus velezensis* B13 as an effective biocontrol agent against *Rhizoctonia solani*, the pathogen responsible for rice sheath blight. Strain B13 exhibited strong antagonistic activity through the production of novel secondary metabolites, mycoparasitic interactions, and induction of plant defense mechanisms. Molecular docking studies further supported the antifungal potential of metabolites with high binding affinity to key fungal pathogenicity proteins. Therefore, *B. velezensis* B13, after successful formulation and field trials across different rice ecosystems, can be used as a potential bio-control agent for the sustainable management of rice sheath blight.

## Data Availability

The original contributions presented in this study are included in the article/**Supplementary Materials**. Further inquiries can be directed to the corresponding author.
