# Supplementary material for: Metabolomic profiling of Bacillus velezensis B13 and unveiling its antagonistic potential for the sustainable management of rice sheath blight
Source: Front Plant Sci. 2025 Jul 25;16:1554867. doi: 10.3389/fpls.2025.1554867 (PMC12331701; doi:10.3389/fpls.2025.1554867)
Supplement: Supplementary file 1 [file DataSheet1.doc]

Supplementary Material

#
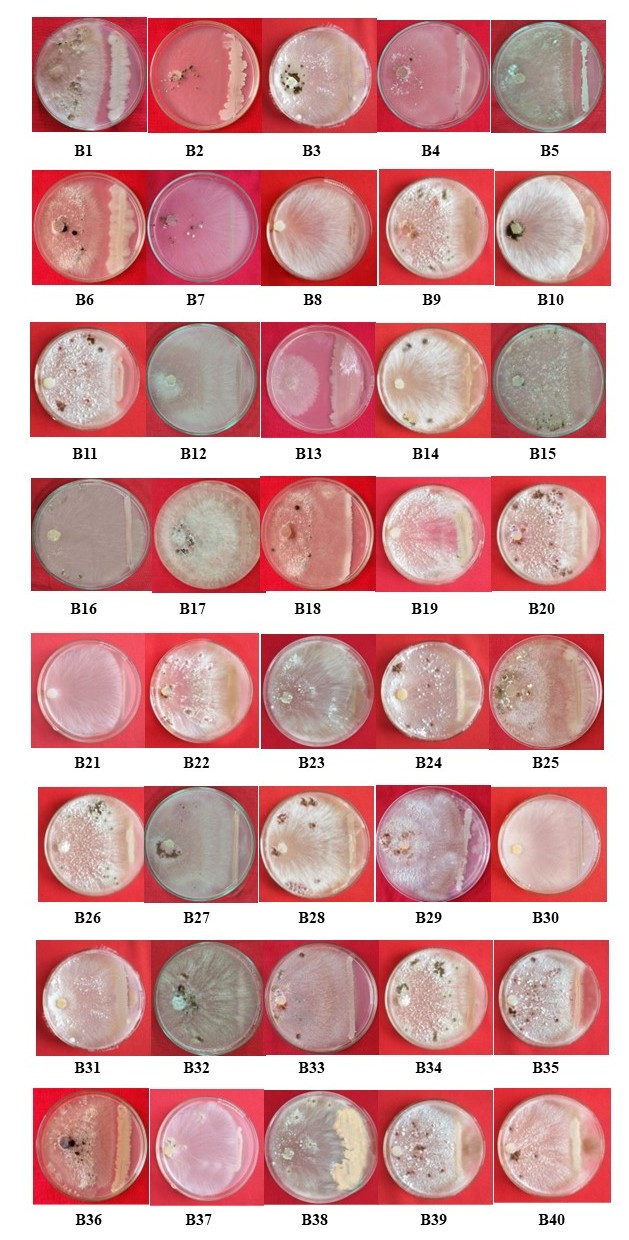


**Figure S1**: Antagonistic potential of rice endophytic bacterial isolates against *R. solani*


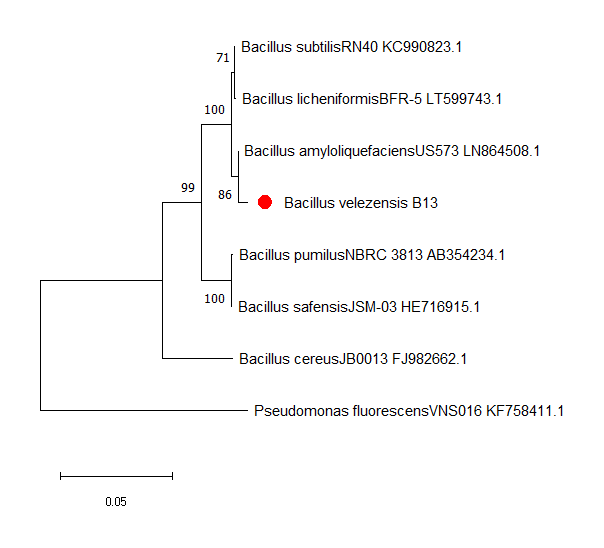


**Figure S2**: Phylogenetic tree generated from 16S-rDNA sequence by using neighbor joining analysis in MEGA 11 software


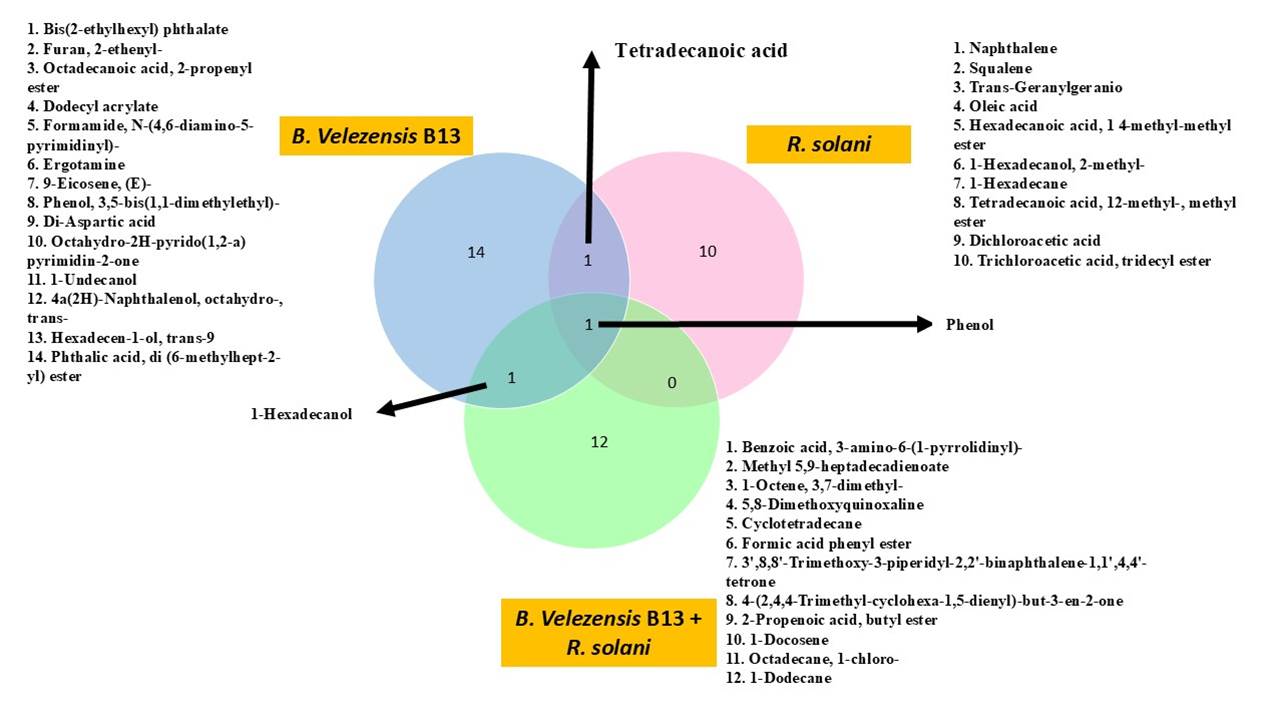


**Figure S3**: Comparative distribution of metabolites produced by *B. velezensis* B13, *B. velezensis* B13 + *R. solani* and *R. solani* alone.

| **A) Actin like protein ARP6** | **Ramachandran Plot for Actin like protein ARP6** |
| --- | --- |
| 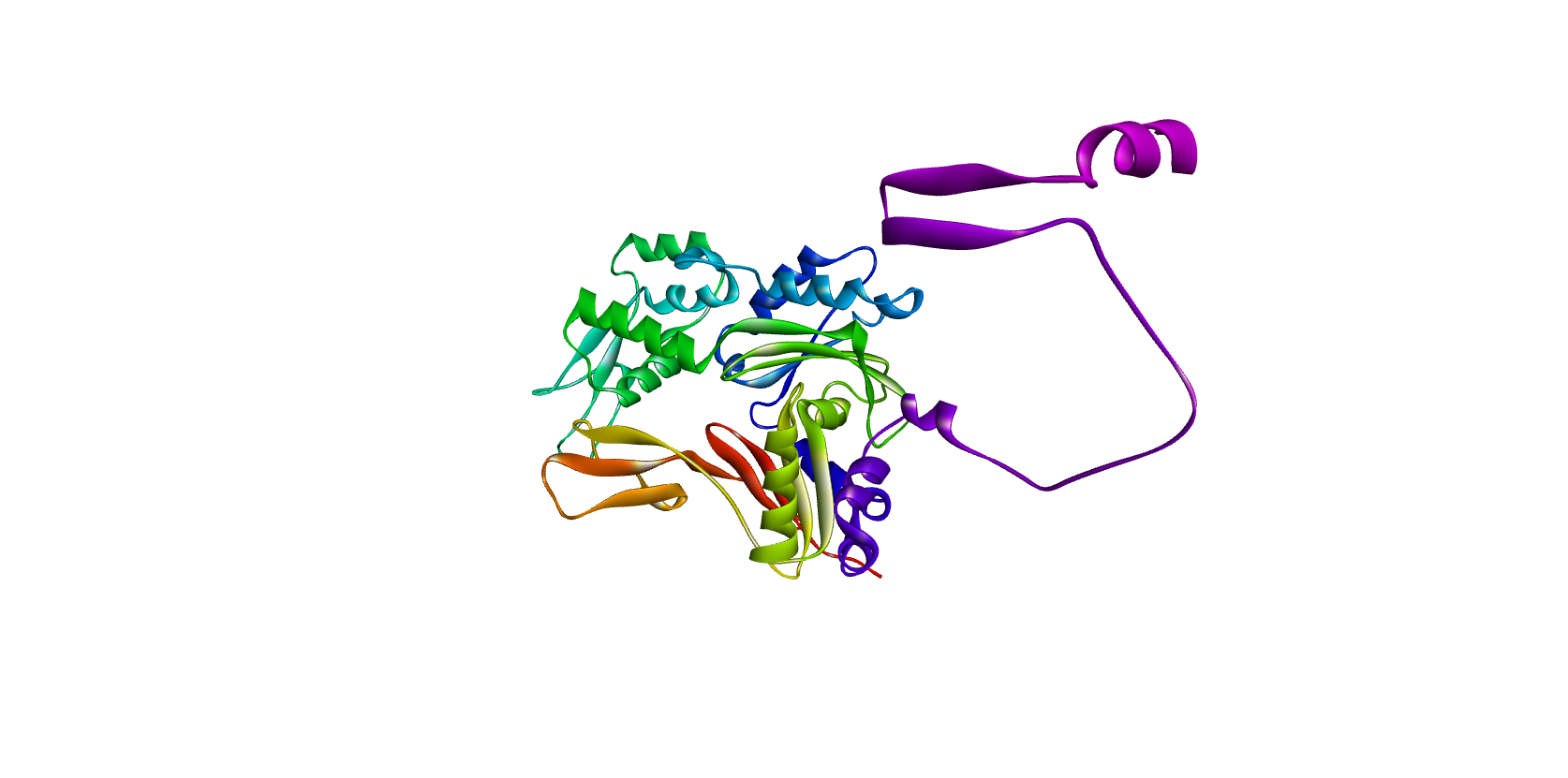 | 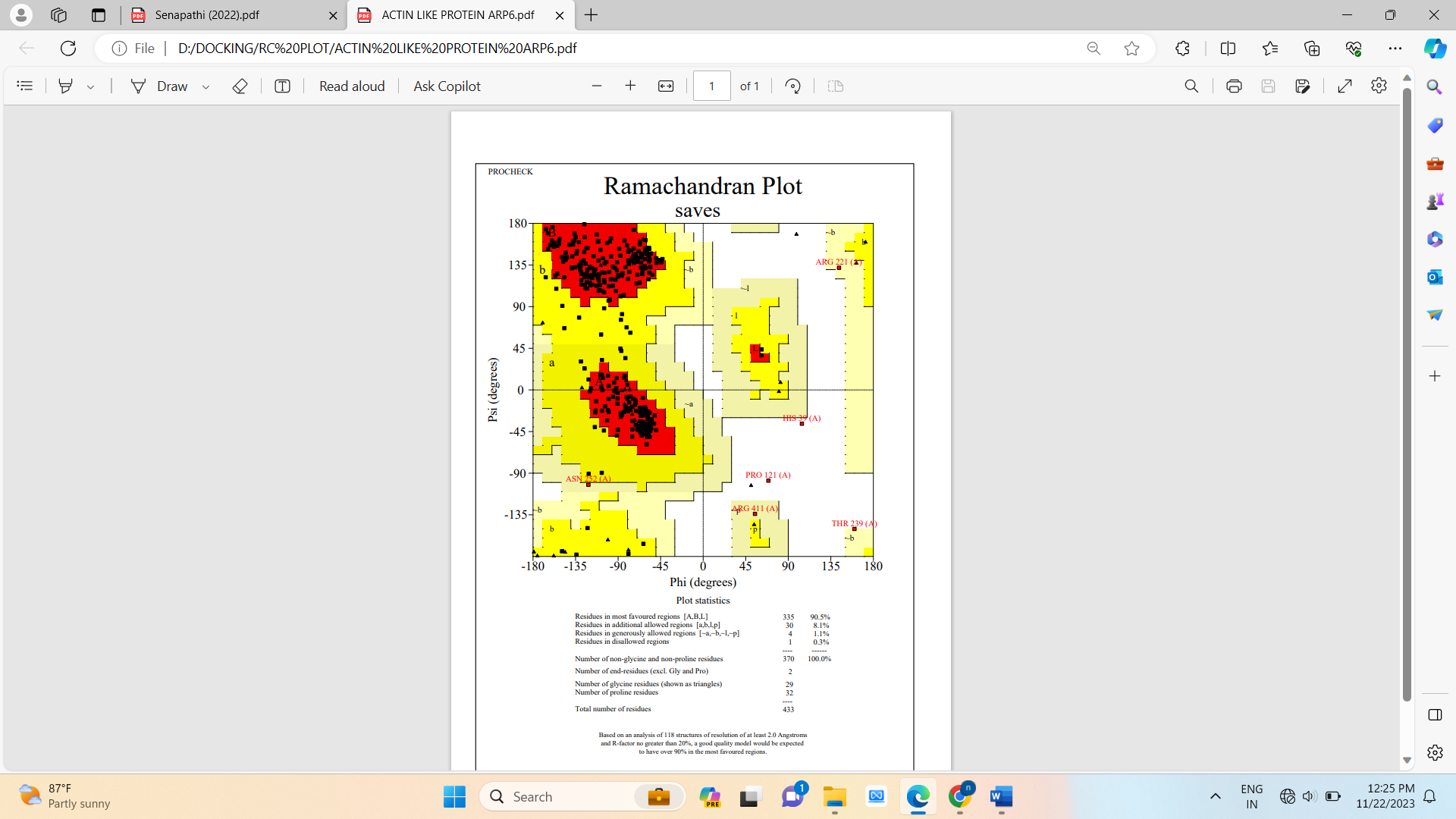 |
| **B) β-1,3 Glucan synthase** | **Ramachandran Plot for β-1,3 Glucan synthase** |
| 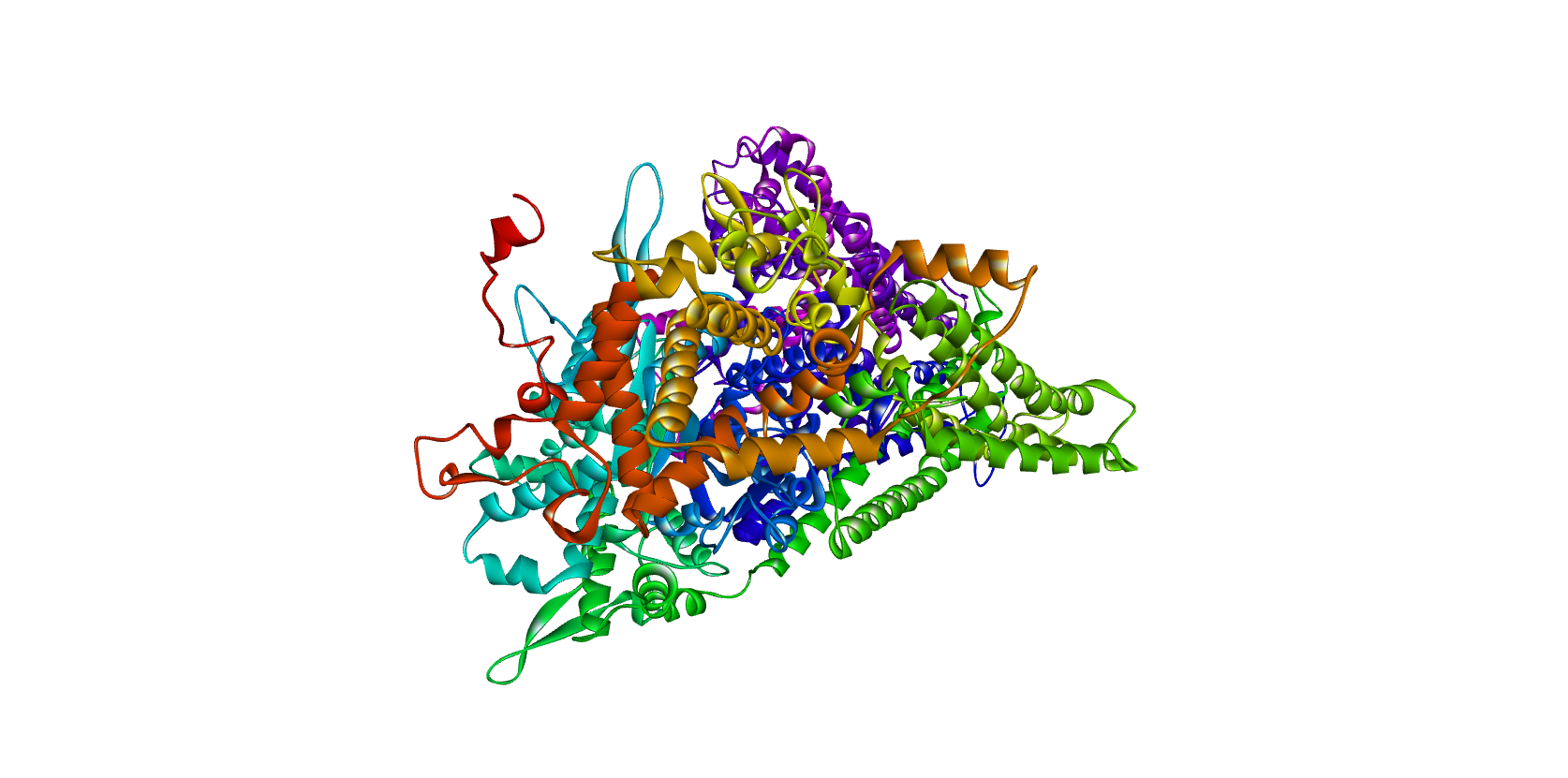 | 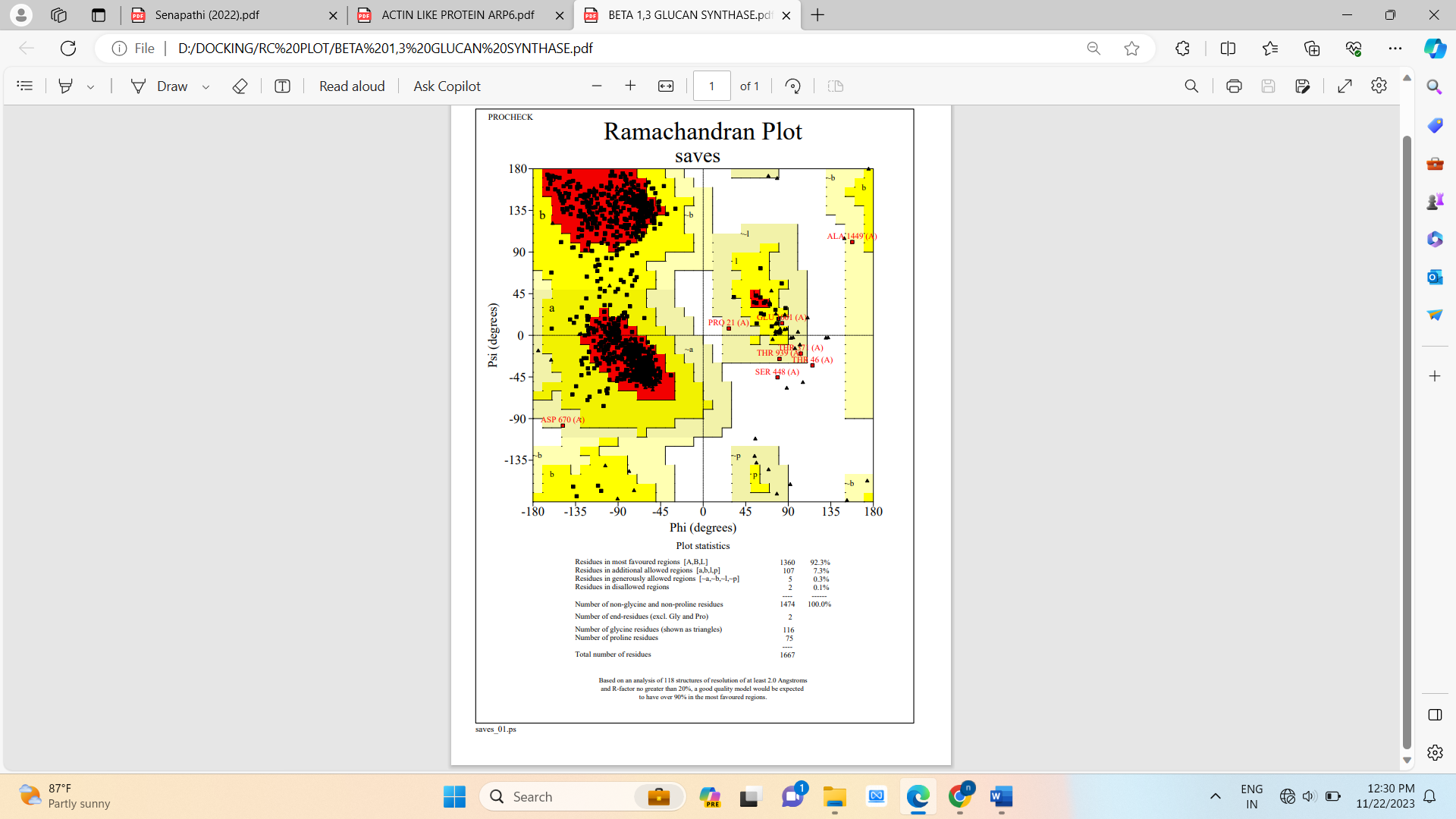 |

| | **C) Pectinesterase** | **Ramachandran Plot for Pectinesterase** | | --- | --- | | 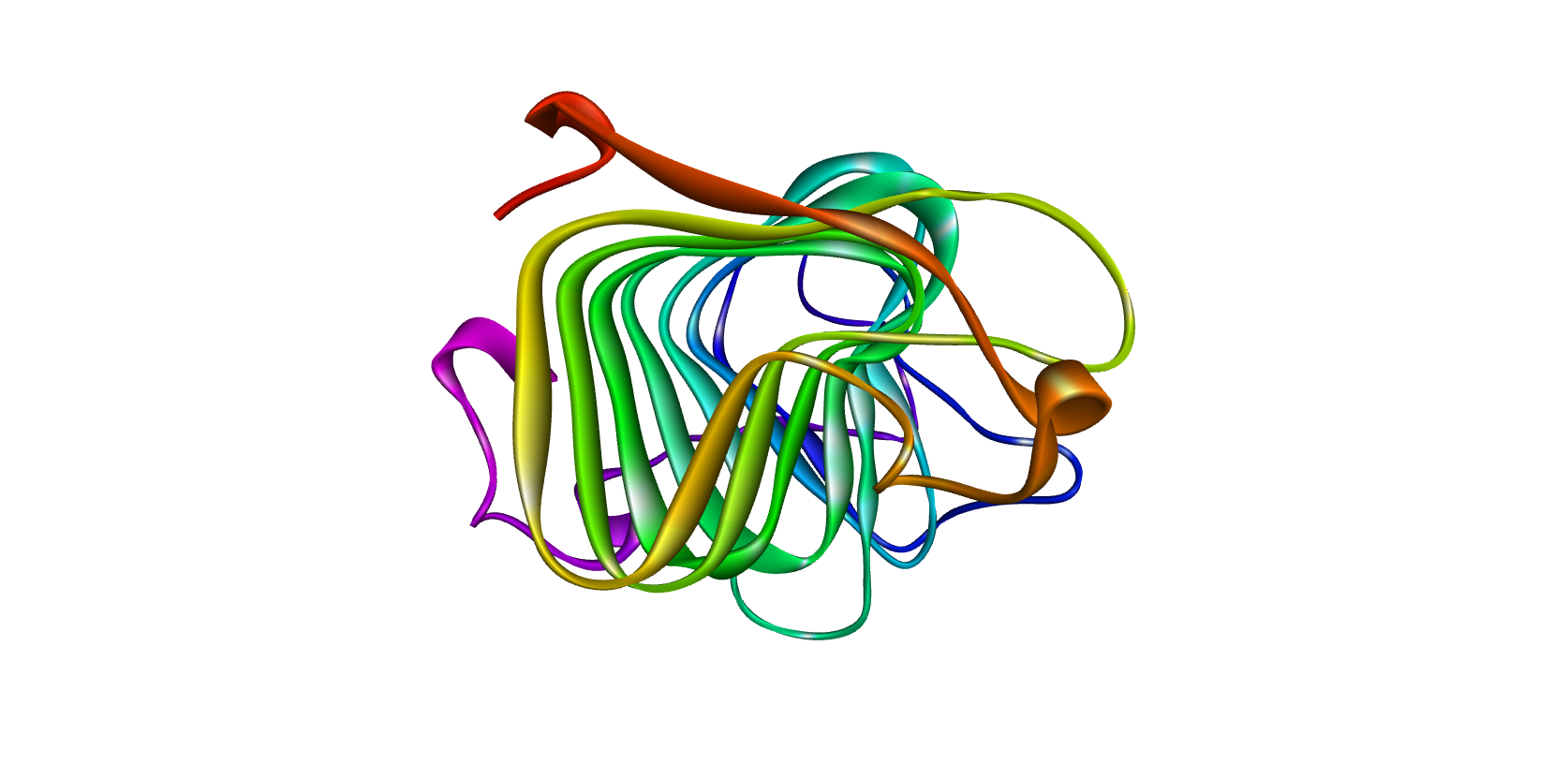 | 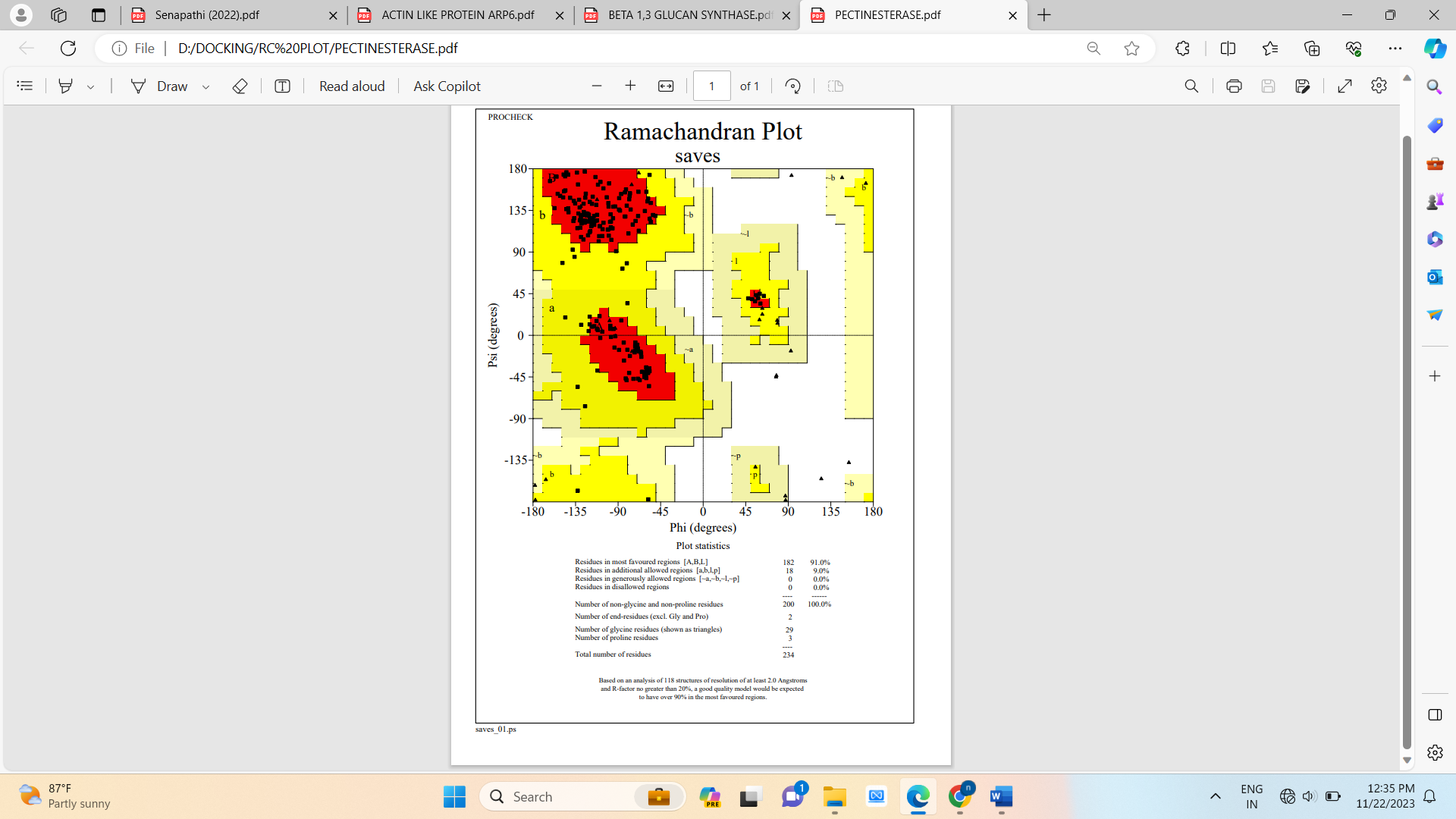 | | **D) Polygalacturonase** | **Ramachandran Plot for Polygalacturonase** | | 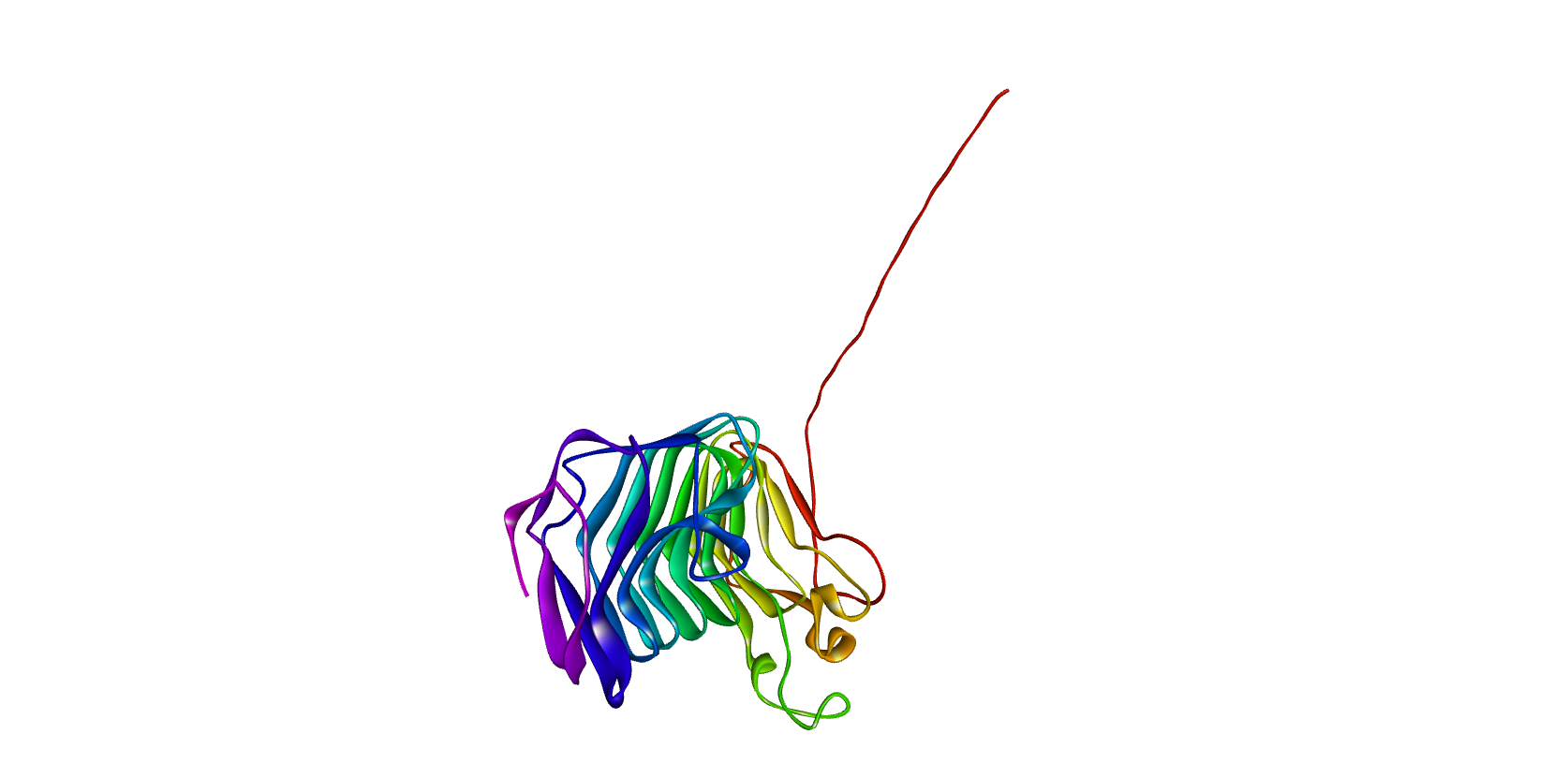 | 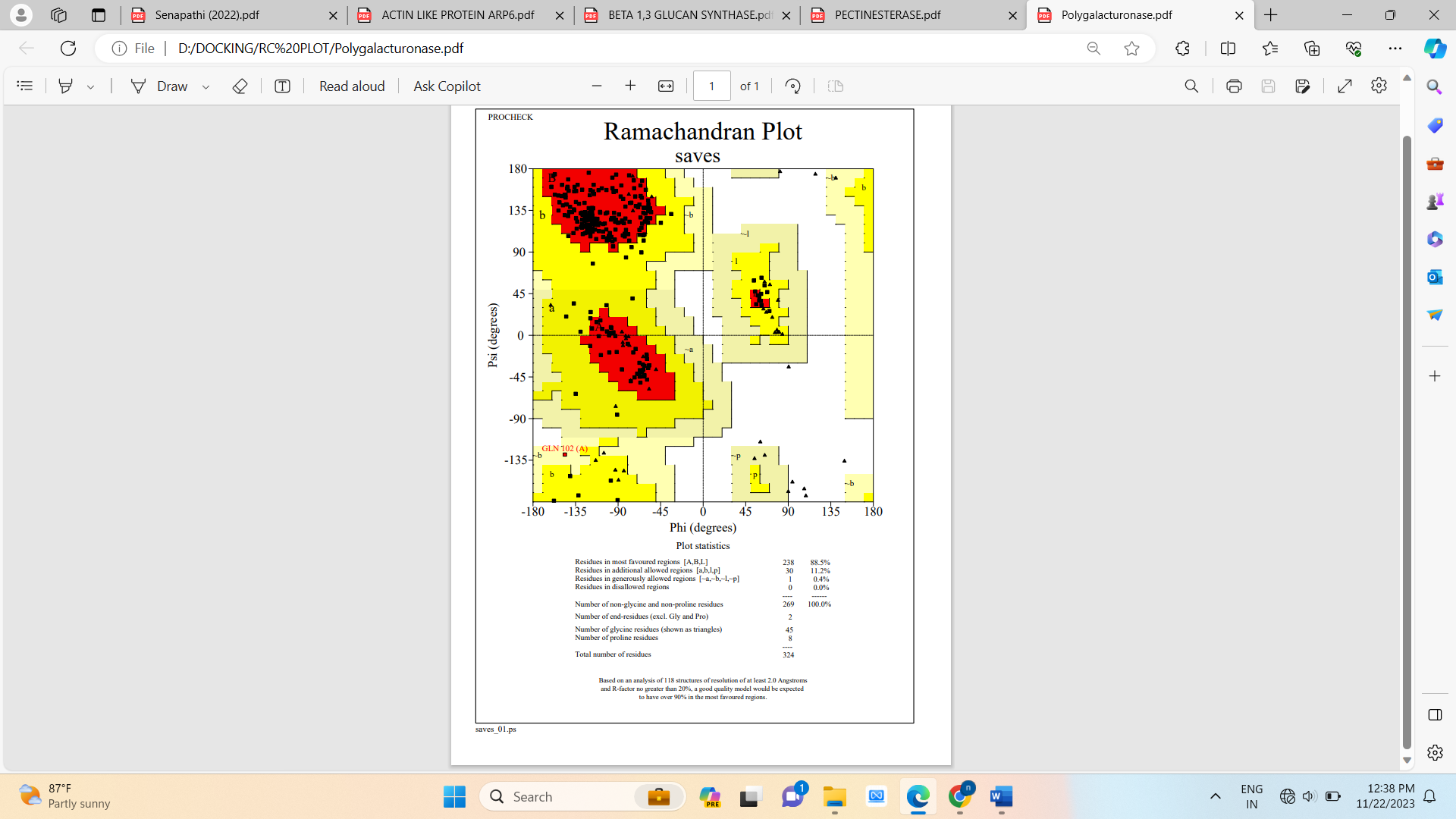 | |  |
| --- | --- | --- | --- | --- | --- | --- | --- | --- | --- |

# Figure S4: Three-dimensional structure representation of protein targets of *R. solani*

**Table S1. Diversity of secondary metabolites produced by *R. solani*** in PDA medium

| **S. no** | **Compound name** | **Retention time** | **Peak Area percentage** | **Molecular weight/Molecular formula** | **Molecular structure** | **Function** | **References** |
| --- | --- | --- | --- | --- | --- | --- | --- |
| 1 | Naphthalene | 7.22 | 8.04 | 128.17  C_10_H_8_ | 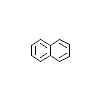 | Antifungal | Boukaew *et al*. (2013) |
| 2 | Squalene | 24.42 | 19.27 | 410.7  C_30_H_50_ | 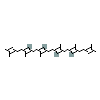 | Antibacterial | Prabhukarthikeyan *et al*. (2014) |
| 3 | Trans-geranylgeraniol | 24.24 | 23.27 | 290.5  C_20_H_34_O | 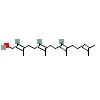 | Antimicrobial | Awan  *et al.* (2023) |
| 4 | Oleic acid | 25.47 | 0.54 | 282.5  C_18_H_34_O_2_ | 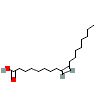 | Antibacterial | Nakkeeran *et al*. (2020) |
| 5 | Hexadecanoic acid, 1 4-methyl-methyl ester | 10.53 | 0.67 | 270.5  C_17_H_34_O_2_ | 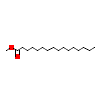 | Antimicrobial  Antibacterial | Abubacker and Deepalakshmi (2013) |
| 6 | 1-Hexadecanol, 2-methyl- | 21.86 | 0.72 | 256.5  C_17_H_36_O | 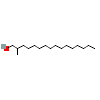 | Antimicrobial | Jayakumar *et al*. (2021) |
| 7 | Phenol | 4.14 | 0.78 | 94.11  C_6_H_5_OH | 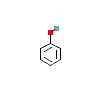 | Antimicrobial | Devi *et al*. (2021) |
| 8 | Tetradecanoic acid, 12-methyl-, methyl ester | 10.26 | 1.95 | 242.40  C_15_H_30_O_2_ | 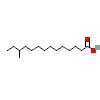 | Antimicrobial  Antioxidant | Dheepa *et al.* (2016) |
| 9 | Dichloroacetic acid | 17.96 | 0.55 | 128.94  C_2_H_2_Cl_2_O_2_ | 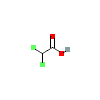 | Antimicrobial | Shobier *et al.* (2016) |
| 10 | Trichloroacetic acid, tridecyl ester | 4.14 | 0.78 | 345.7  C_15_H_27_Cl_3_O_2_ | 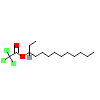 | Antimicrobial | Sholkamy *et al*. (2023) |
| 11 | Tetradecanoic acid | 5.01 | 0.92 | 228.37  C_14_H_28_O_2_ | 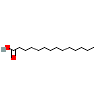 | Antibacterial | Dheepa *et al*. (2016) |
| 12 | 1-Hexadecane | 29.46 | 1.15 | 226.44  C_16_H_34_ | 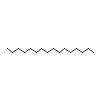 | Antimicrobial | Dheepa *et al.* (2016) |

**Table S2. Diversity of secondary metabolites produced by *B. velezensis*** B13

| **S. no** | **Compound name** | **Retention time** | **Peak Area percentage** | **Molecular weight/Molecular formula** | **Molecular structure** | **Function** | **References** |
| --- | --- | --- | --- | --- | --- | --- | --- |
| 1 | Bis(2-ethylhexyl) phthalate | 16.28 | 0.77 | 128.17  C_10_H_8_ | 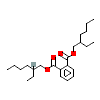 | Antifungal | Abd Ali *et al*. (2023) |
| 2 | Octahydro-2H-pyrido(1,2-a) pyrimidin-2-one | 9.98 | 4.09 | 154.21  C_8_H_14_N_2_O | 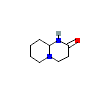 | Antimicrobial | - |
| 3 | 1-Undecanol | 20.66 | 2.64 | 172.31  C_11_H_24_O | 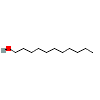 | Antibacterial | [Li](javascript:void(0)) *et al*. (2020) |
| 4 | 4a(2H)-Naphthalenol, octahydro-, trans- | 17.18 | 12.76 | 154.25  C_10_H_18_O | 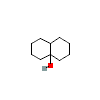 | Antifungal | Ali *et al*. (2021) |
| 5 | Hexadecen-1-ol, trans-9 | 13.82 | 4.69 | 240.42  C_16_H_32_O | 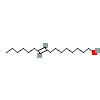 | Antibacterial | [Hidayathulla](https://pubmed.ncbi.nlm.nih.gov/?term=Hidayathulla+S&cauthor_id=29356238) *et al*. (2018) |
| 6 | Furan, 2-ethenyl- | 16.97 | 16.24 | 94.11  C_6_H_6_O | 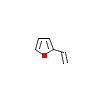 | Antifungal | Poveda, (2021) |
| 7 | Phthalic acid, di (6-methylhept-2-yl) ester | 17.96 | 3.32 | 390.6  C_24_H_38_O_4_ | 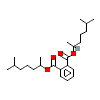 | Antibacterial | Zhang *et al*. (2011) |
| 8 | Octadecanoic acid,2-propenyl ester | 15.25 | 2.18 | 324.5  C_21_H_40_O_2_ | 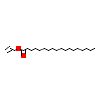 | Antifungal | Nakkeeran *et al*. (2020) |
| 9 | Dodecyl acrylate | 18.41 | 1.46 | 240.38  C_15_H_28_O_2_ | 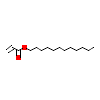 | Antimicrobial | Grahovac and Vlajkov (2023) |
| 10 | Formamide, N-(4,6-diamino-5-pyrimidinyl)- | 20.31 | 1.26 | 153.14  C_5_H_7_N_5_O | 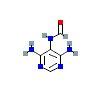 | Antibacterial | _ |
| 11 | Ergotamine | 21.88 | 1.68 | 581.7  C_33_H_35_N_5_O_5_ | 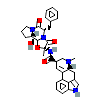 | Antifungal | Conrado *et al.* (2022) |
| 12 | 9-Eicosene (E) | 7.21 | 0.81 | 280.5  C_20_H_40_ | 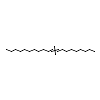 | Antimicrobial | Singh *et al*. (2021) |
| 13 | Phenol, 3,5-bis(1,1-dimethylethyl)- | 15.92 | 0.96 | 206.32  C_14_H_22_O | 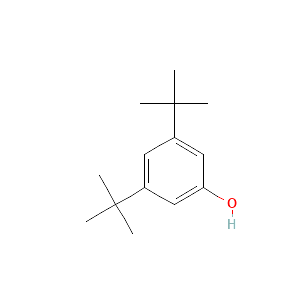 | - | - |
| 14 | Di-Aspartic acid | 3.40 | 0.79 | 133.1  C_4_H_7_NO_4_ | 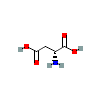 | Antibacterial | - |

**Table S3. Diversity of secondary metabolites produced in the axenic culture of *B. velezensis* B13 and its interaction with
*R. solani*** in PDA medium

| **S. no** | **Compound name** | **Retention time** | **Peak Area percentage** | **Molecular weight/Molecular formula** | **Molecular structure** | **Function** | **References** |
| --- | --- | --- | --- | --- | --- | --- | --- |
| 1 | Benzoic acid, 3-amino-6-(1-pyrrolidinyl)- | 3.16 | 0.72 | 137.14  C_7_H_7_NO_2_ | 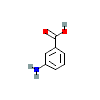 | Antifungal | [Karthik](https://pubmed.ncbi.nlm.nih.gov/?term=Karthik Y%5BAuthor%5D) *et al.* (2023) |
| 2 | Methyl 5,9-heptadecadienoate | 3.31 | 0.82 | 266.4  C_17_H_30_O_2_ | 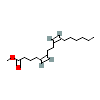 | Antifungal | Devakumar *et al.*  (2017) |
| 3 | 1-Octene, 3,7-dimethyl- | 7.21 | 2.35 | 140.27  C_10_H_20_ | 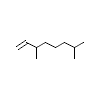 | Antimicrobial | - |
| 4 | 5,8-Dimethoxyquinoxaline | 13.81 | 3.84 | 158.20  C_10_H_10_N_2_ | 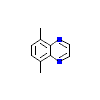 | Antimicrobial | [Tang](https://pubs.rsc.org/en/results?searchtext=Author%3AXuemei Tang) *et al.* (2022) |
| 5 | Cyclotetradecane | 3.40 | 0.95 | 196.37  C_14_H_28_ | 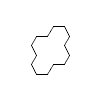 | Antifungal | Kumari *et al.* (2019) |
| 6 | Formic acid phenyl ester | 4.15 | 1.33 | 122.12  C_7_H_6_O_2_ | 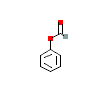 | Antimicrobial | Koilybayeva *et al*. (2023) |
| 7 | 3',8,8'-Trimethoxy-3-piperidyl-2,2'-binaphthalene-1,1',4,4'-tetrone | 7.21 | 2.35 | 487.5  C_28_H_25_NO_7_ | 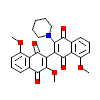 | Antifungal | - |
| 8 | 4-(2,4,4-Trimethyl-cyclohexa-1,5-dienyl)-but-3-en-2-one | 15.24 | 4.50 | 190.28  C_13_H_18_O | 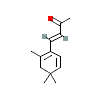 | Antibacterial | - |
| 9 | 2-Propenoic acid, butyl ester | 3.01 | 3.55 | 128.17  C_7_H_12_O_2_ | 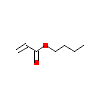 | Antifungal | Mujeeb *et al*. (2014) |
| 10 | 1-Docosene | 25.48 | 1.24 | 308.6  C_22_H_44_ | 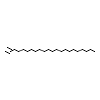 | Antibacterial | Bughio *et al*. (2017) |
| 11 | Octadecane, 1-chloro- | 15.92 | 1.05 | 288.9  C_18_H_37_Cl | 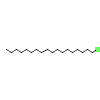 | Antimicrobial | Sharma *et al*. (2024) |
| 12 | 1-Dodecane | 21.88 | 1.79 | 170.3  C_12_H_26_ | 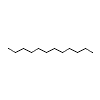 | Antifungal | Ponnusamy *et al*., (2018) |
| 13 | 1-Hexadecanol | 17.96 | 3.43 | 242.4  C_16_H_32_O | 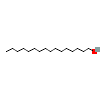 | Antibacterial | - |

**Table S4: Pathogenicity/ virulence protein targets of *R. solani* used for molecular docking**

| **S. No.** | **Name of the protein targets** | **Role** | **Reference** | **Uniprot Id** |
| --- | --- | --- | --- | --- |
| 1 | Actin like protein ARP_6_ | Structure and function of nucleolus. Role in transcriptional regulation of fungus | Prabhukarthikeyan *et al*. (2022) | A0A8H3CCY1 |
| 2 | Polygalacturonase PG3 | Induce sheath tissue necrosis and release sugar for pathogenicity. Act as virulence factor | Chen *et al*. (2017) | L8X539 |
| 3 | Pectinesterase | Involved in plant cell wall degradation and an important pathogenicity factor | Bhaskar rao *et al*., (2020) | L8X224 |
| 4 | β 1,3-glucan synthase | Aid in adhesion to host plant and contribute the evasion of plant defenses. Essential for successful invasion and disease progression. | Zheng *et al*. (2013) | L8WXK6 |

**Table S5: Quality assessment of modelled protein targets of *R.solani* through SWISS Modelling server**

| **S. No.** | **Name of the protein targets** | **Sequence identity** | **Sequence similarity** | **Coverage** | **Gmqe** | **Residues in allowed region** |
| --- | --- | --- | --- | --- | --- | --- |
| 1 | Actin like protein ARP_6_ | **71.36%** | **0.53** | **1.00** | **0.82** | **99.7%** |
| 2 | Polygalacturonase PG3 | **90.91%** | **0.58** | **0.98** | **0.93** | **99.9%** |
| 3 | Pectinesterase | **73.93%** | **0.53** | **1.00** | **0.98** | **100%** |
| 4 | β 1,3-glucan synthase | **70.16%** | **0.53** | **0.96** | **0.81** | **100%** |
